# Early recruitment of membrane-bound DNaseX to phagocytic cups in macrophages

**DOI:** 10.7554/eLife.110907

**Published:** 2026-10-08

**Authors:** Arghajit Pyne, Vivek Pandey, Subhankar Kundu, Sachie Ikegami, Xuefeng Wang

**Affiliations:** 1 Hoxworth Center, College of Medicine, University of Cincinnati Cincinnati United States; 2 Department of Pathology and Laboratory Medicine, College of Medicine, University of Cincinnati Cincinnati United States; University of Sao Paulo Brazil; Universitat Pompeu Fabra Spain

**Keywords:** phagocytosis, nuclease activity, surface nuclease sensor, macrophages, neutrophil extracellular trap, biofilm, Human

## Abstract

Macrophages engulf and degrade pathogens and cellular debris through phagocytosis. The degradation process was generally believed to occur only after phagosome internalization and maturation. Here, we report an early DNase activity at the nascent phagocytic cup (PC) prior to its closure. Using a fluorescent DNase sensor, we revealed rapid and ubiquitous DNase activity upon PC formation across various macrophage types. We further identified the responsible enzyme as the membrane-bound DNaseX, which is constitutively recruited to the PC during PC formation. F-actin polymerization was found to correlate with DNase activity in the PC, likely by promoting physical engagement of DNaseX with solid DNA materials. Functionally, we show that macrophages degrade extracellular DNA (eDNA) within bacterial biofilms through direct physical contact, clearing the eDNA structures without internalization. These findings reveal a previously unrecognized DNA degradation mechanism operating at the macrophage membrane, suitable for degrading bulky eDNA materials which cannot be directly internalized by macrophages.

## Introduction

Professional phagocytes, including neutrophils, macrophages, and dendritic cells, are crucial for immune defense and homeostasis maintenance. These cells perform phagocytosis to ingest and break down pathogens, as well as senescent and dead self-cells (Aderem, 2003; Gordon, 2016; Inomata et al., 2020; Underhill and Ozinsky, 2002). During phagocytosis, phagocytes form actin-rich, cup-shaped invaginations of the plasma membrane that envelop target particles (Swanson, 2008). These phagocytic cups (PCs) are subsequently closed at the cup rims through actin polymerization and myosin-mediated constriction (Vorselen et al., 2021), leading to the internalization of particles into vacuoles known as phagosomes (Freeman and Grinstein, 2014; Hallett, 2020). Phagosomes undergo maturation by fusing with endosomes and lysosomes, from which phagosomes acquire various hydrolases such as nucleases, proteases, and lipases (Fountain et al., 2021; Mylvaganam and Freeman, 2023; Uribe-Querol and Rosales, 2020; Wong et al., 2017; Yates et al., 2005). These hydrolases degrade a variety of biomolecules that make up pathogens and self-cells, playing a critical role in pathogen elimination and biomaterial recycling. In particular, among these hydrolases, DNases play a crucial role in breaking down pathogenic and self-cell-generated extracellular DNA (eDNA) (He et al., 2009; Samejima and Earnshaw, 2005; Schuermans et al., 2024), which can cause inflammation (Kowarsky et al., 2017), severe autoimmune diseases (Bai et al., 2018; Lachowicz-Scroggins et al., 2019), and organ damage (Cahilog et al., 2020; Czaikoski et al., 2016) if not cleared promptly.

Traditionally, it is believed that DNA degradation during phagocytosis begins only after phagosome-lysosome fusion, during which the phagosome acquires DNase II and becomes acidic, creating optimal conditions for DNase II activity (Evans and Aguilera, 2003). However, this ‘digest-after-ingest’ mechanism likely limits macrophages from utilizing phagocytosis to degrade bulky DNA materials, e.g., in neutrophil extracellular traps (NETs) (Papayannopoulos, 2018; Kaplan and Radic, 2012) or bacterial biofilms (Whitchurch et al., 2002). Both are composed of eDNA structures that exceed the size of phagocytes and are therefore unlikely to be internalized by macrophages in their entirety. Although DNase I and Caspase-activated DNase (Napirei et al., 2004; Nagata, 2005) have been proposed to degrade eDNA materials, these soluble DNases could be rapidly diluted in body fluids and may lack the capacity to achieve the localized, concentrated degradation needed to effectively break down solid eDNA released by pathogens or self-cells.

In this study, we investigated the occurrence of DNase activity during phagocytosis and discovered an unexpected early onset of DNase activity within the PC even before its closure. A fluorescent surface-immobilized nuclease sensor (SNS) (Pal et al., 2021; Wang et al., 2019), previously developed in our lab, was adopted to coat microbeads, which bait the phagocytosis of macrophages. SNS, essentially as a DNA construct, emits fluorescent signals upon its degradation, faithfully reporting on-site DNase activity at the cell-particle interface. We found that DNase activity is ubiquitously strong within the PC irrespective of the sizes of phagocytic targets (polystyrene microbeads of different sizes) or macrophage types. A series of inhibition and immunostaining results established that the DNase in the PC is glycosylphosphatidylinositol (GPI)-anchored membrane-bound DNaseX, which is also known as DNase I-like 1 (DNase1L1) (Shiokawa et al., 2007). The recruitment of DNaseX to the PC was shown to be constitutive, responding to both microbeads and pathogens (here *Escherichia coli*), regardless of the immunogenic material present on the microbeads. We have also demonstrated that macrophages directly degrade eDNA structures in the *Staphylococcus aureus*-formed biofilm by physical contact. The early DNase activity in the PC defines a new interaction paradigm between macrophages and eDNA structures. This ‘digest-before-ingest’ mechanism may greatly empower macrophages to efficiently degrade large-sized eDNA structures in NETs and biofilms using localized and concentrated DNaseX on the cell membrane.

## Results

### Strong DNase activity in the unclosed PC

**Figure 1.**
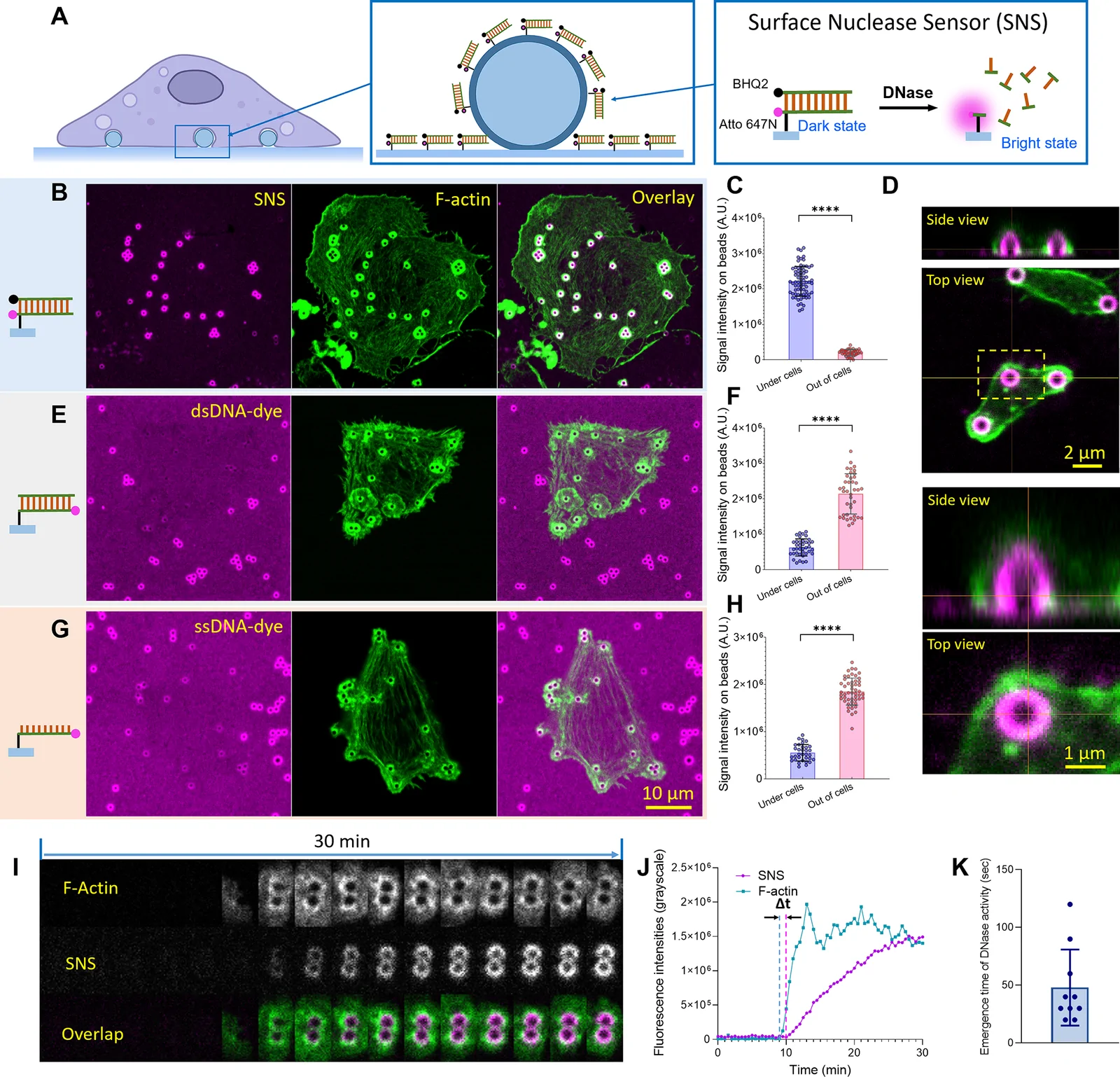
Early onset of DNase activity in the phagocytic cup (PC) prior to its closure. (**A**) Surface-immobilized nuclease sensor (SNS) reports DNase activity in the PCs of macrophages. Microbeads are immobilized on a glass substrate to elicit phagocytosis. The entire surface, including the microbeads, is coated with SNS, a double-stranded DNA (dsDNA) labeled with a quencher-dye pair. SNS becomes fluorescent upon degradation by DNase. (**B**) Human THP-1 macrophages were plated on the DNase-reporting platform, where fluorescence signals appeared in ring patterns, co-localizing with F-actin surrounding the microbeads. (**C**) Comparison of SNS fluorescence intensities on microbeads underneath cell bodies versus those outside of cells. (****p<0.0001; Each data point represents one PC; n=3 experiments; error bars indicate SD.) (**D**) Confocal 3D scanning revealed that the SNS signal was localized on the surface of microbeads. (**E, F**) dsDNA-dye coated on microbeads exhibited a decrease in fluorescence intensities beneath macrophages. (**G, H**) Single-stranded DNA (ssDNA)-dye coated on microbeads exhibited a decrease in fluorescence intensities beneath macrophages. (**I**) Time-lapse co-imaging of F-actin and SNS signals in the PCs of live macrophages. (**J**) Signal intensity curves of F-actin and SNS on one microbead. The time gap Δt between the starting times of these two signals is defined as the emergence time of DNase activity in the PCs. (**K**) Emergence time of DNase activity in the PCs was statistically estimated to be 48±33 s after the PC formation indicated by F-actin signal. Figure 1—source data 1.Numeric data and statistical analysis for the plots in Figure 1 and associated figure supplements.

We developed a platform that induces macrophage phagocytosis and enables the visualization of DNase activity in the PC through fluorescence imaging. As shown in Figure 1A, polystyrene microbeads were deposited on a glass surface, and SNS was coated on both the substrate and microbeads. The SNS construct is an 18 base-paired double-stranded DNA (dsDNA) decorated with a quencher (blackhole quencher 2), an Atto647N dye, and a biotin tag facilitating the surface immobilization of the SNS (Figure 1A) through biotin-avidin interaction. The initially non-fluorescent SNS, if degraded by DNase, leaves a dye freed from quenching on the surface, thereby reporting the local DNase activity by fluorescence on the cell-particle or cell-substrate interfaces. The functionality of SNS on a glass surface was validated by monitoring its fluorescence response to soluble DNase I treatment (Figure 1—figure supplement 1).

**Figure 1—figure supplement 1.**
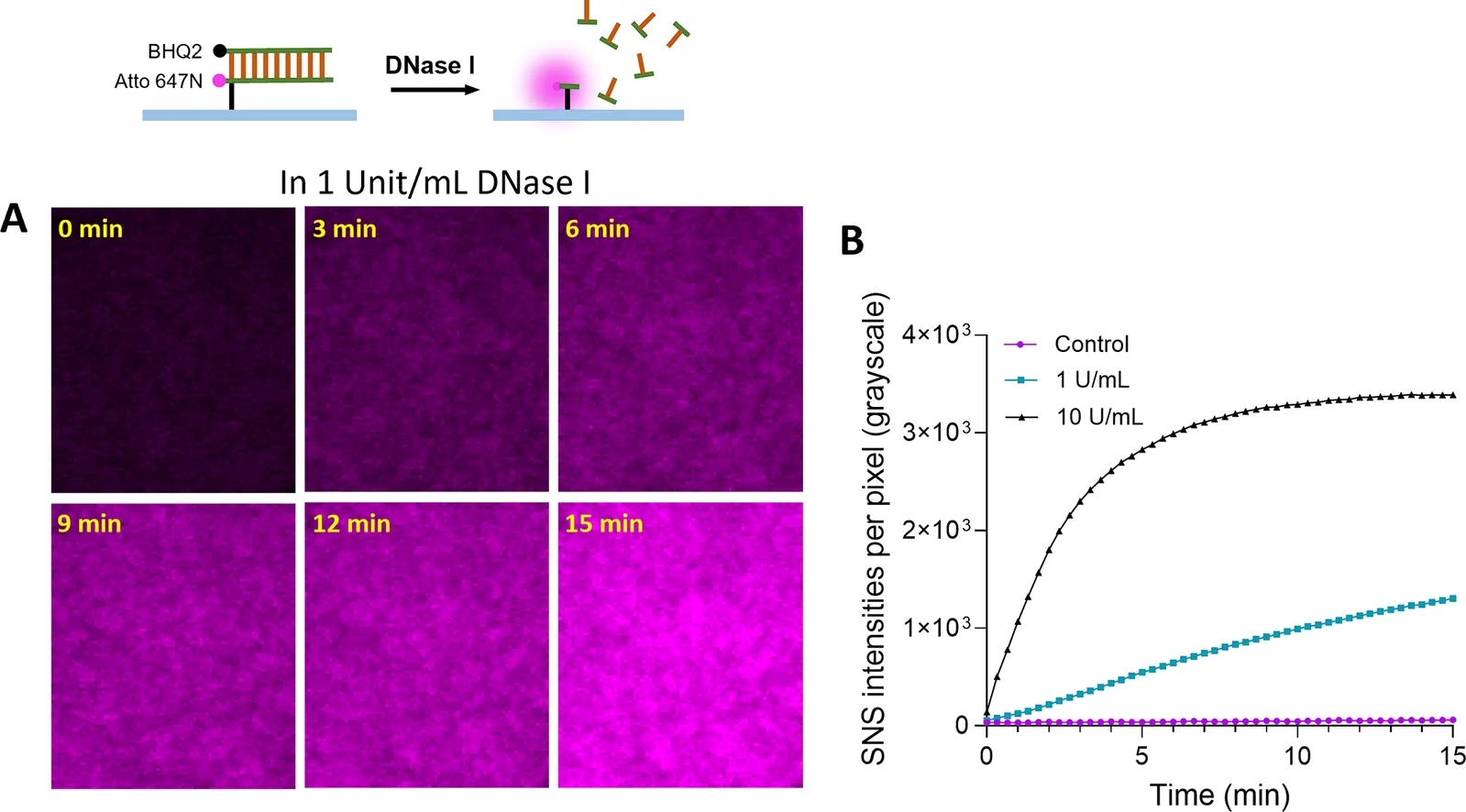
Surface-immobilized nuclease sensor (SNS) immobilized on glass surfaces responds to the treatment of soluble DNase I. (**A**) 1 U/mL DNase I (Thermo Fisher Scientific, #89836) in Tris buffer (10 mM Tris-HCl, 2.5 mM MgCl_2_, 0.5 mM CaCl_2_, pH 7.5) was loaded on an SNS-coated glass surface. Fluorescence images were acquired as a time series. (**B**) SNS response curves to the treatment of DNase I at concentrations of 0 (control), 1 U/mL, and 10 U/mL.

**Figure 1—figure supplement 2.**
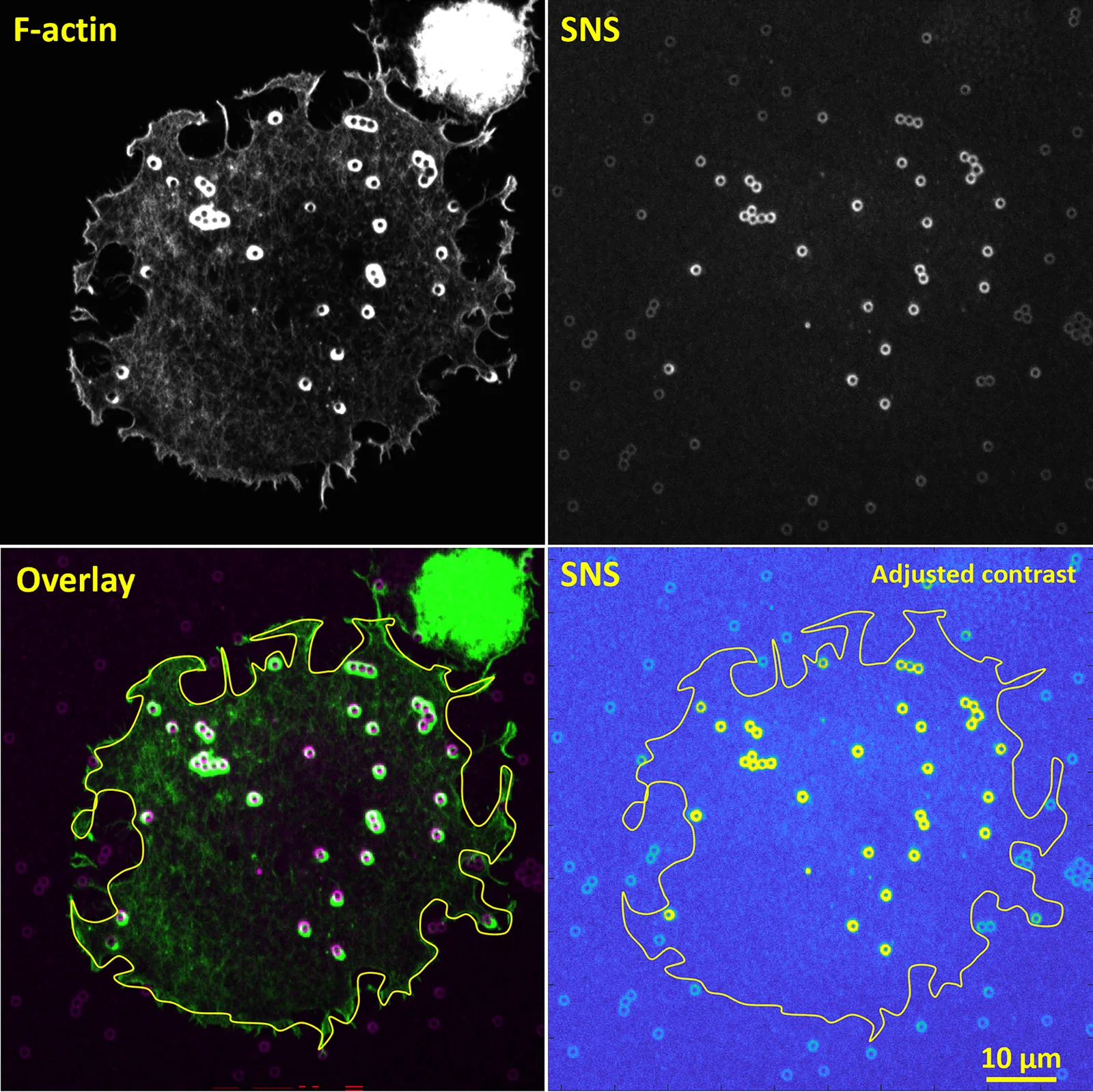
Surface-immobilized nuclease sensor (SNS) signals on microbeads specifically occurred underneath macrophage cell bodies. The SNS sensor displays weak fluorescence despite the quencher in the sensor construct for the non-100% quenching efficiency. This low background fluorescence was leveraged to visualize all microbeads on the glass surface by digitally enhancing the image contrast. It was confirmed that the SNS signals, indicated by localized increases in fluorescence, appeared exclusively on the microbeads located beneath the cell bodies of THP-1 macrophages. This observation reinforces the notion that the DNase activity indicated by SNS signals is specific to the cell regions. The yellow contour represents the cell’s edge.

**Figure 1—figure supplement 3.**
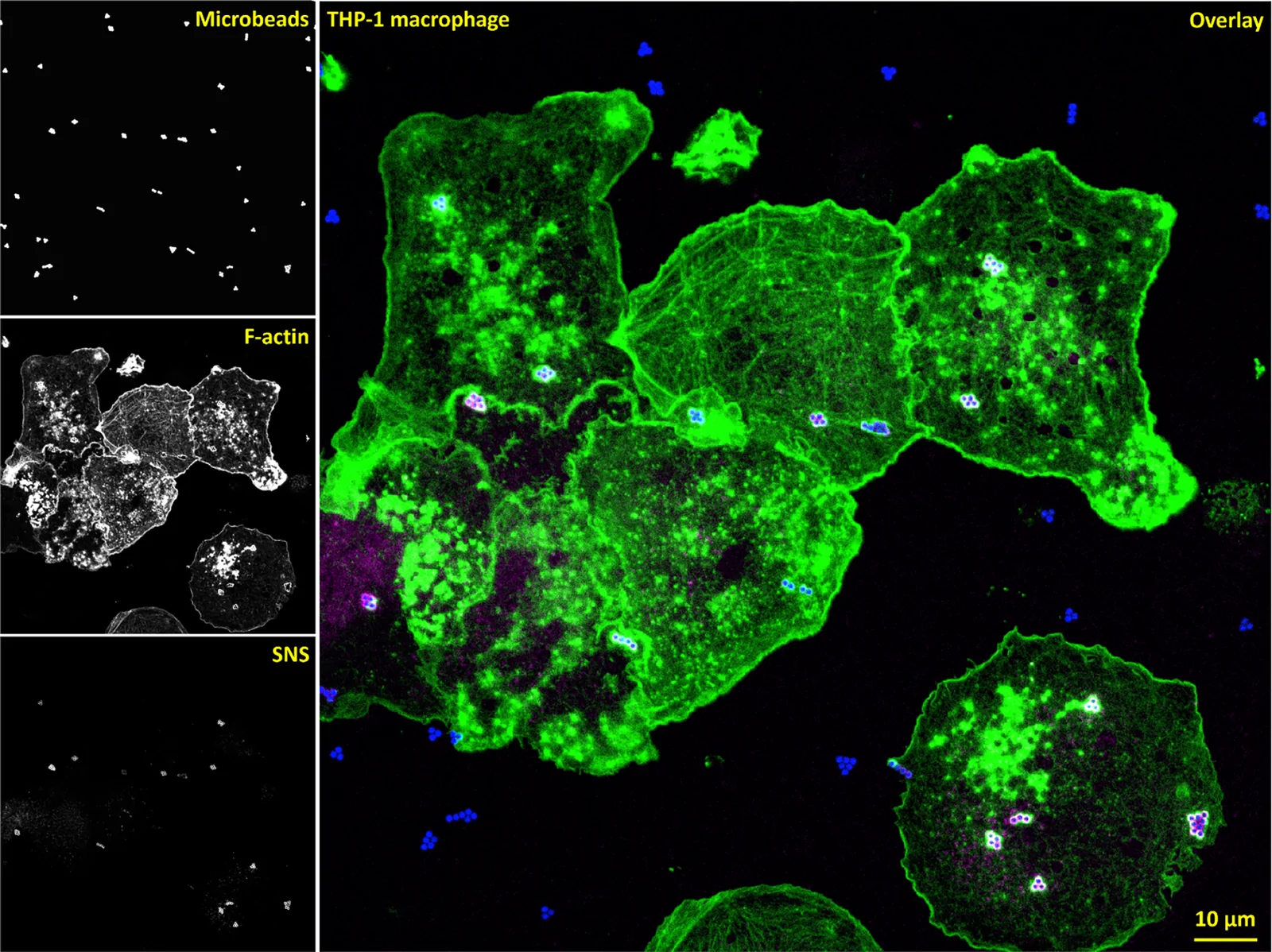
Large-area imaging of THP-1 macrophages on a surface-immobilized nuclease sensor (SNS) surface. Fluorescent beads with an excitation peak at 405 nm were used in SNS experiments to facilitate their localization during fluorescence imaging. Large-area imaging was performed over human THP-1 macrophages plated on an SNS-coated surface, showing microbeads (λ=405 nm, blue), F-actin (λ=488 nm, green), and SNS-reported DNase activity (λ=647 nm, magenta). The macrophages were incubated on the surface for 1 hr prior to cell fixation and staining. These images demonstrate that THP-1 macrophages specifically and consistently produce DNase activity in phagocytic cups.

**Figure 1—figure supplement 4.**
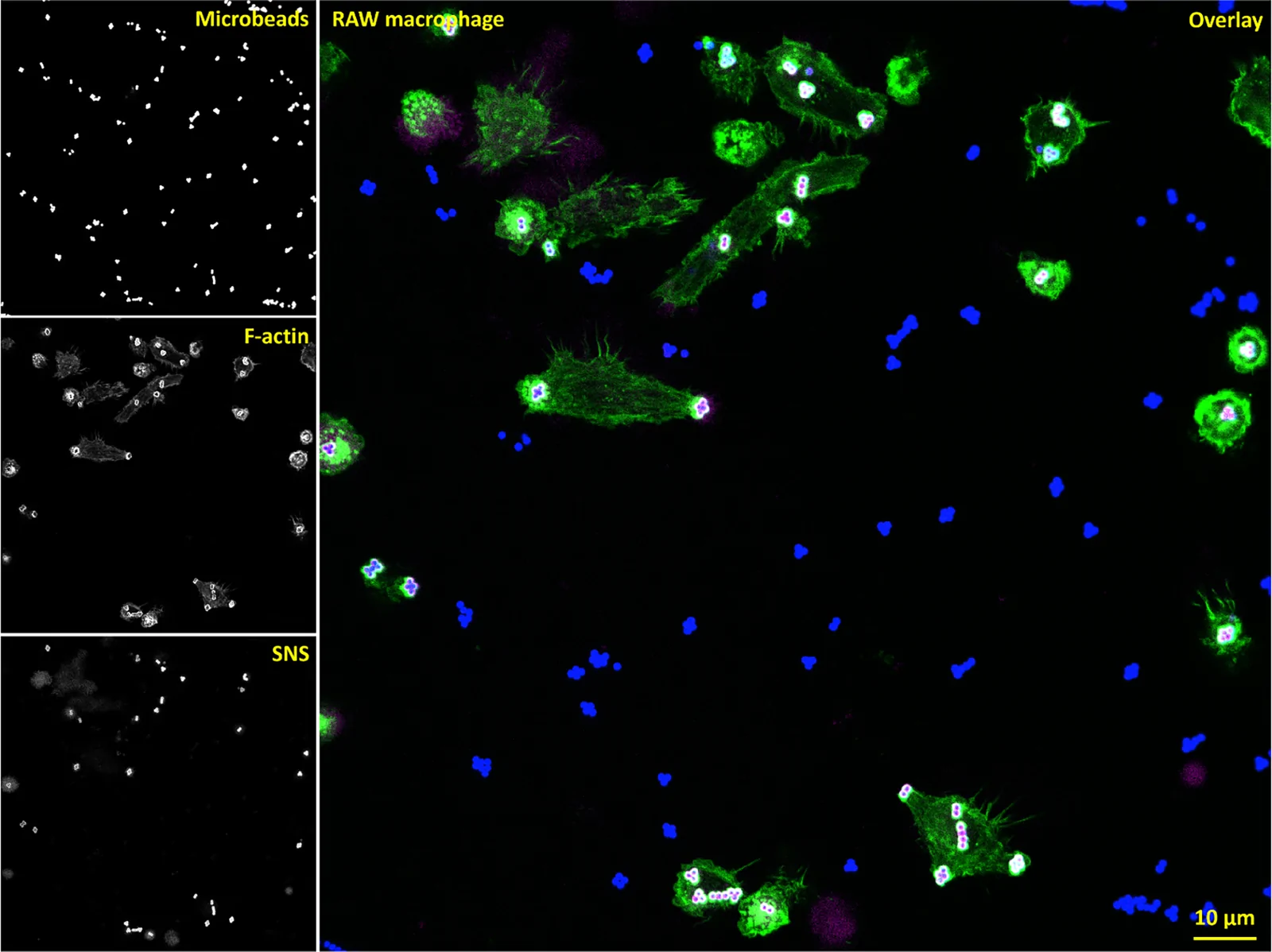
Large-area imaging of RAW macrophages on a surface-immobilized nuclease sensor (SNS) surface. Large-area imaging was performed over mouse RAW 264.7 macrophages plated on an SNS-coated surface, showing microbeads (λ=405 nm, blue), F-actin (λ=488 nm, green), and SNS-reported DNase activity (λ=647 nm, magenta). The macrophages were incubated on the surface for 1 hr prior to cell fixation and staining. These images demonstrate that RAW macrophages specifically and consistently produce DNase activity in phagocytic cups.

**Figure 1—figure supplement 5.**
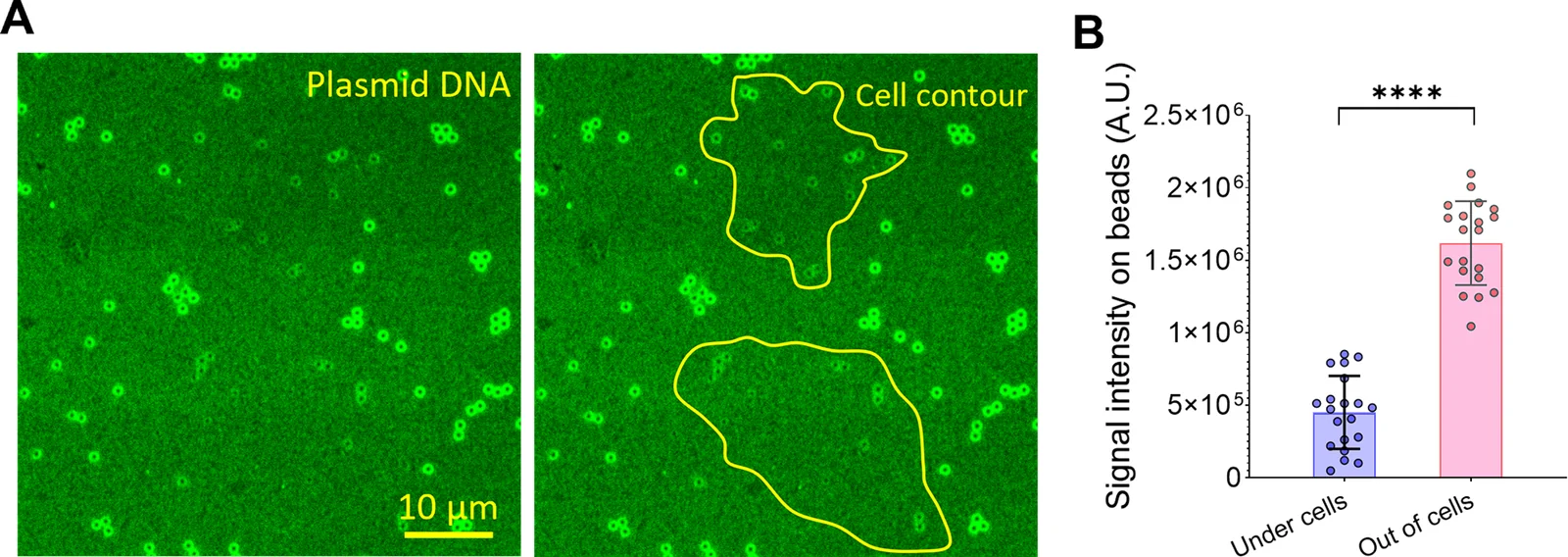
The DNase in phagocytic cups (PCs) degrades plasmid DNA. (**A**) 50 µg/mL plasmid DNA was immobilized on a microbead-decorated and poly-L-lysine (PLL)-coated glass surface. The immobilization of plasmid DNA was enabled by the electrostatic force as the PLL coating is positively charged and the DNA backbone is negatively charged. The DNA was stained with SYTOX Green. RAW macrophages were incubated on the surface for 1 hr. The sample was directly imaged without cell fixation or staining. (**B**) Fluorescence intensities of SYTOX Green on microbeads. The microbeads underneath cells had lower fluorescence intensities than those out of cells, suggesting that PCs degrade plasmid DNA as well.

**Figure 1—figure supplement 6.**
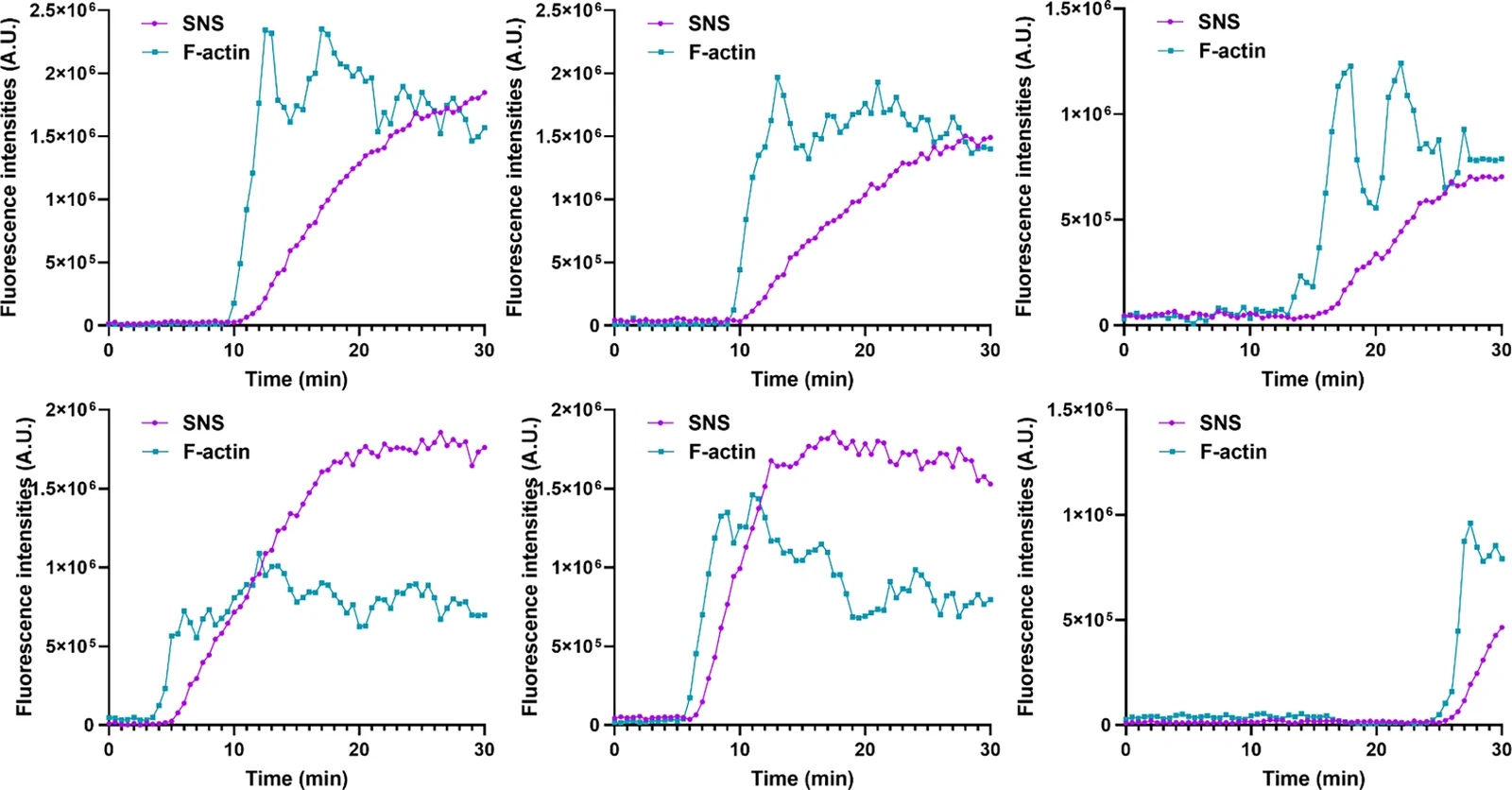
F-actin and surface-immobilized nuclease sensor (SNS) signal intensities in individual phagocytic cups (PCs). F-actin and SNS signal intensity curves from six additional PCs. Imaging was performed in live macrophages transfected with LifeAct-GFP and plated on SNS-coated surfaces. Related to Video 1.

**Figure 1—figure supplement 7.**
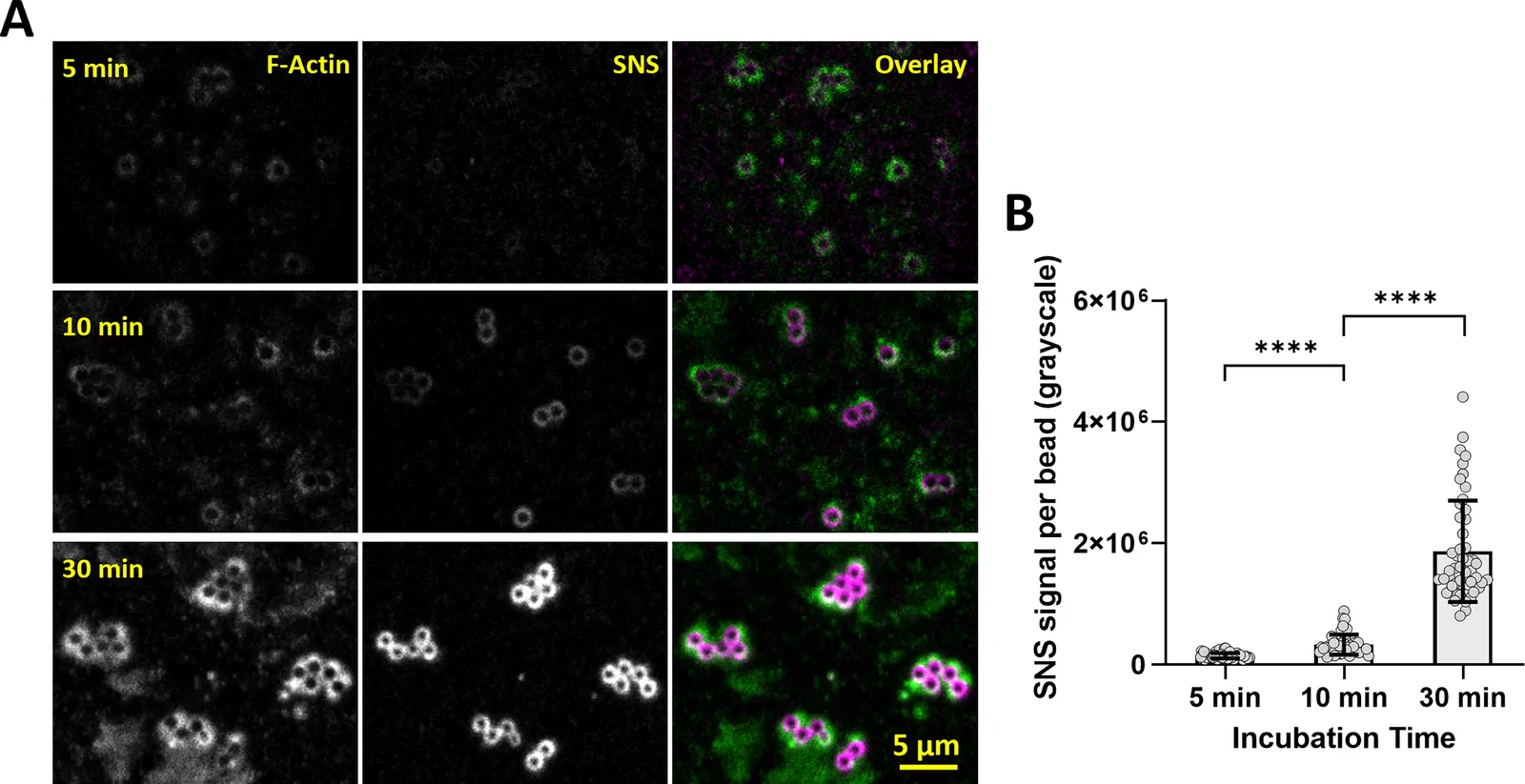
Surface-immobilized nuclease sensor (SNS) signal intensities in the phagocytic cups (PCs) after cell plating for 5, 10, and 30 min, respectively. (**A**) Co-imaging of F-actin and SNS in the PCs of THP-1 macrophages with 5, 10, and 30 min cell incubation times. (**B**) Quantification of the SNS signal intensities. Each data point represents the total fluorescent intensity of the SNS signal on a microbead. ****p<0.0001. Each data point represents the SNS signal in one PC.

**Figure 1—figure supplement 8.**
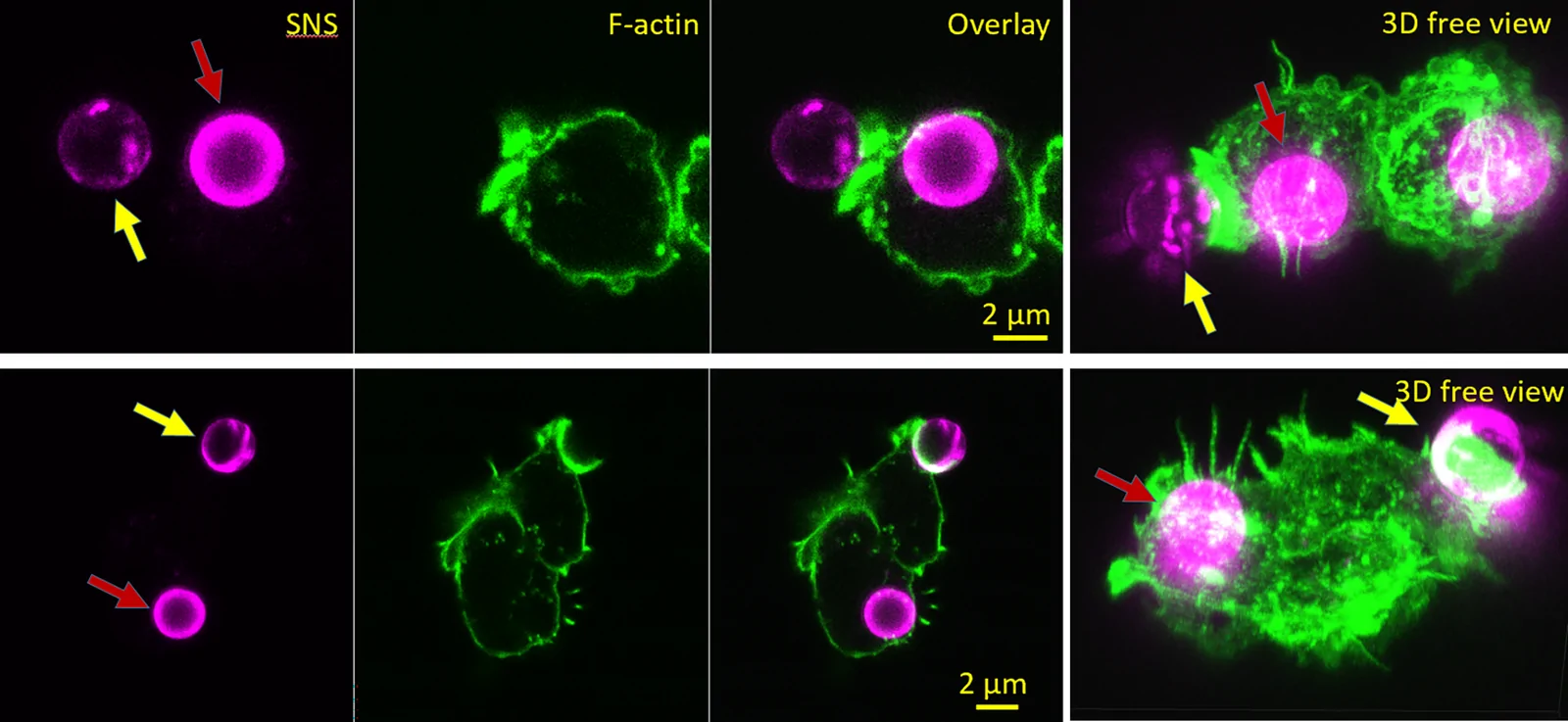
The phagocytic cup (PC) exhibits DNase activity over free microbeads (not surface-immobilized). Streptavidin-functionalized microbeads (3 µm) were coated with surface-immobilized nuclease sensor (SNS) via the avidin-biotin interaction and then added to surface-adherent RAW macrophages. After 30 min of incubation, many microbeads (red arrows) had been internalized by the macrophages and exhibited strong fluorescence, indicating DNase activity within the phagosomes. Several microbeads appeared to be undergoing phagocytosis, which is not yet completed and were co-localized with PCs, as indicated by F-actin-rich cup structures. Notably, these microbeads (yellow arrows) displayed non-uniform fluorescence across their surfaces, suggesting that PCs already have DNase activity in response to free microparticles before PC closure.

Next, human THP-1 macrophages were plated on the microbead-decorated and SNS-coated surface. After incubation for 30 min, the cells were then fixed and F-actin was stained to evaluate the formation of PCs. Fluorescence imaging revealed circular F-actin structures specifically surrounding microbeads, suggesting that macrophages formed PCs in response to the microbeads. Remarkably, strong SNS fluorescence signals, signifying local DNase activity, were observed around the microbeads beneath the macrophages, co-localizing with the circular F-actin signals (Figure 1B). Note that many SNS-coated microbeads were also present outside of the cell regions, and those microbeads showed no SNS fluorescence signals (Figure 1C, Figure 1—figure supplement 2). Moreover, SNS was coated on the entire platform, including both the surfaces of the microbeads and the substrate. However, only the regions of the PCs displayed strong SNS signals, suggesting that SNS signals were highly specific to the PCs formed by the macrophages. To further confirm the specificity and consistency of DNase activity in PCs, large-area imaging was performed on macrophages cultured on fluorescent microbead-decorated and SNS-coated surfaces. The results show that nearly all PCs within macrophages exhibited strong SNS signals, whereas microbeads located outside the cells did not, as shown in Figure 1—figure supplements 3 and 4.

While SNS signals appear as ring-shaped patterns around the microbeads in two-dimensional imaging, confocal three-dimensional scanning revealed that these signals were actually generated across the entire surface of the microbeads underneath the domes of the PCs, rather than on the substrate regions surrounding the beads (Figure 1D). To rule out the possibility that the SNS signals in the PCs resulted from dsDNA dissociation due to other factors, such as helicases (albeit unlikely), we further assessed DNase activity in the PCs using dye-labeled dsDNA as a DNase sensor (Figure 1E). This construct is expected to report DNase activity by fluorescence loss upon degradation and should not respond to dsDNA dissociation. This strategy of detecting enzymatic degradation through fluorescence loss is similar to the conventional dye-labeled gelatin degradation assay used to evaluate the protease activity of invadosomes (Berdeaux et al., 2004). The result indeed showed that the fluorescence loss was specifically associated with the PCs over the microbeads under THP-1 macrophage cell bodies (Figure 1E), reinforcing the notion that the dsDNA construct of the SNS was degraded rather than being dissociated within PCs. The average fluorescence loss on microbeads within the PCs is greater than 70%, indicating that the majority of dsDNA was degraded within the PCs (Figure 1F). Additionally, we tested dye-labeled single-stranded DNA (ssDNA) as a DNase sensor and found that the PCs also caused fluorescence loss of ssDNA on microbeads, suggesting that the DNase in the PCs is capable of degrading ssDNA as well (Figure 1G and H).

We further demonstrated that DNase within the PCs is capable of degrading long dsDNA. As shown in Figure 1—figure supplement 5, plasmid DNA extracted from *E. coli* was immobilized on a microbead-decorated glass surface through physical adsorption. The plasmid DNA was stained with SYTOX Green. Macrophages were then plated onto the surface and incubated for 1 hr. Fluorescence intensities of SYTOX Green (DNA stain) associated with microbeads underneath cells were significantly lower than those of microbeads outside the cells, indicating that the plasmid DNA immobilized on the microbeads was degraded by macrophages.

These results revealed strong and specific DNase activity in the PCs of macrophages that degrades both dsDNA and ssDNA, a phenomenon that had not been previously recognized. Because SNS reports DNase activity by fluorescence gain, which has higher specificity and sensitivity than using fluorescence loss, we used SNS as the DNase sensor in the following experiments.

### DNase activity occurs within 1 min after the initiation of PC formation

We demonstrated that DNase activity in macrophage PCs occurs prior to cup closure. To further investigate the timing of this DNase activity during PC formation, we performed live-cell imaging of F-actin and SNS signals during phagocytosis. Cells were transfected with LifAact-GFP, a fluorescent peptide that binds F-actin and enables its visualization in live cells. Due to the high transfection resistance of THP-1 cells, we used mouse RAW 264.7 macrophages as a model for transfection and live imaging.

As shown in Figure 1I and Video 1, F-actin and SNS signals were imaged in separate fluorescence channels, with the F-actin signal appearing slightly earlier than the SNS signal. Signal intensity analysis in Figure 1J indicates that the time gap between the onset of these two signals is approximately 1 min. Statistical evaluation based on 10 PCs determined this time gap to be 48±33 s (Figure 1K, Figure 1—figure supplement 6), suggesting that DNase activity generally occurs within 1 min after the initiation of PC formation. This timing is considerably earlier than the usual duration required for phagosomes to acquire DNase II through phagosome formation, maturation, and fusion with lysosomes, a process that typically takes tens of minutes to hours (Canton et al., 2014; Peña-Ramos et al., 2022).

**Video 1.** Co-imaging of surface-immobilized nuclease sensor (SNS) signals and F-actin in phagocytic cups. The cells are RAW macrophages transfected with LifeAct-GFP. The actual duration of cell activity in the video is 30 min.

We also assessed the time required for DNase activity to be detectable in PCs after cell plating. As shown in Figure 1—figure supplement 7, SNS signals on the microbeads were clearly visible within 10 min of cell plating. This result reinforces the notion that macrophages quickly initiate DNase activity in response to phagocytic targets.

Because surface-immobilized microbeads prevent PC closure, they likely prolong PC formation and may alter the observed temporal dynamics of DNase activity within the PC. To determine whether the PC exhibits DNase activity before closure, we prepared SNS-coated free microbeads and fed them to adherent macrophages. The results showed that PCs indeed exhibited DNase activity before cup closure in response to free microbeads (Figure 1—figure supplement 8), confirming that DNase activity is initiated rapidly during PC formation.

### DNase activity occurred in the PCs of all tested macrophages

**Figure 2.**
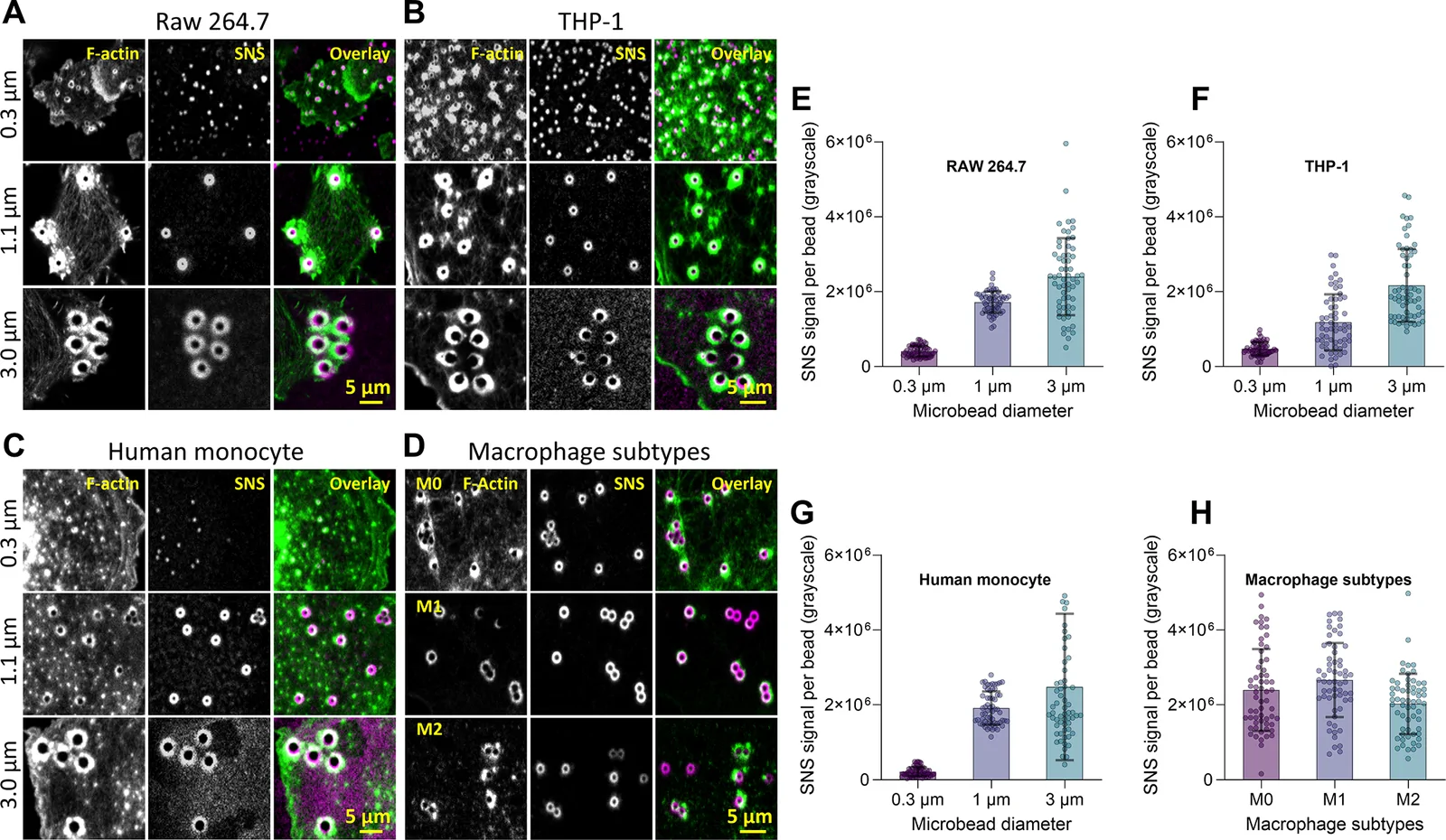
DNase activity is universally present in the phagocytic cups (PCs) of various macrophage types. (**A–C**) DNase activities were consistently observed in the PCs of mouse RAW macrophages, human THP-1 macrophages, and human monocyte-derived macrophages. The PCs, marked by F-actin structures, were formed on microbeads of various sizes (0.3, 1.1, and 3.0 µm). (**D**) DNase activity was consistently observed in the PCs of M0, M1, and M2 THP-1 macrophage subtypes. (**E–G**) Surface-immobilized nuclease sensor (SNS) signal intensities in the PCs of the three types of macrophages. (Each data point represents one PC; n=3 experiments; error bars indicate SD.) (**H**) SNS signal intensities in the PCs of the three subtypes of THP-1 macrophages. (Each data point represents one PC; n=3 experiments; error bars indicate SD.) Figure 2—source data 1.Numeric data and statistical analysis for the plots in Figure 2.

After demonstrating DNase activity in the PCs, we proceeded to investigate the ubiquity of this activity across different cell types and phagocytic particle sizes. We tested three types of macrophages: mouse RAW264.7 macrophages, human THP-1 macrophages, and human macrophages derived from monocytes isolated from peripheral blood (Figure 2—figure supplement 1). Cells of each type were plated on substrates decorated with microbeads of sizes 0.3, 1.1, and 3.0 µm, with both the substrates and microbeads coated with SNS. These cells were incubated for 1 hr and fixed for F-actin staining and cell imaging. In all cases, PCs were observed to form on microbeads, indicated by F-actin structures (Figure 2A–C). SNS signals were consistently and specifically generated on the microbeads within PCs, irrespective of the bead sizes and cell types. The SNS signal intensities were analyzed and plotted in Figure 2E–G. These data suggest that DNase activity is ubiquitous in the PCs.

**Figure 2—figure supplement 1.**
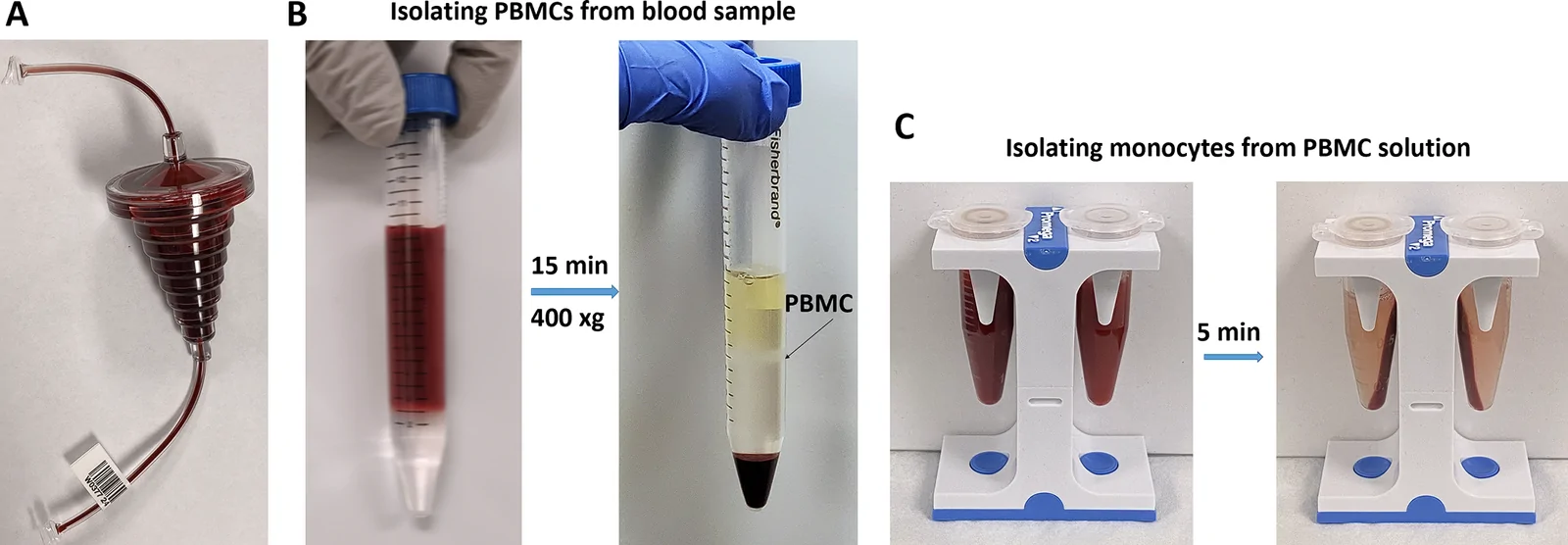
Human monocyte extraction from white blood cell (WBC)-enriched blood sample. (**A**) A leukocyte reduction system (LRS) chamber used during blood donation was collected. The chamber contains WBC-enriched blood sample, providing a source for extracting human monocytes. The blood sample was drained into a 50 mL tube. Phosphate-buffered saline (PBS) supplemented with 2 mM ethylenediaminetetraacetic acid (EDTA) and 2% fetal bovine serum (FBS) was added to the blood sample at a volumetric ratio of 3:1 (PBS:blood). (**B**) 6 mL diluted blood sample was loaded on top of 3 mL gradient medium (the colorless transparent liquid at the bottom of the tube) carefully, forming a clear line between two liquids. The sample was centrifuged at 400×*g* for 15 min. Consequently, red blood cells were spun to the bottom, and peripheral blood mononuclear cells (PBMCs) were concentrated between the plasma (the pinkish top layer of liquids) and the gradient medium. The PBMCs were collected by pipetting and suspended in 2 mL PBS supplemented with 2 mM EDTA and 2% FBS. (**C**) Monocytes in the PBMC solution were isolated using a negative selection kit. This kit employs antibody-coated paramagnetic beads to capture all non-monocyte cells, leaving monocytes in the solution. The purified monocytes were subsequently centrifuged and suspended in culture medium for cell culture and experimentation, or, alternatively, suspended in a solution of 20% dimethyl sulfoxide (DMSO) and 80% FBS for cryogenic preservation.

Dependent on their origins and functions, macrophages generally have three subpopulations: naïve macrophages (referred to as M0 macrophages), pro-inflammatory macrophages (M1 macrophages), and anti-inflammatory macrophages (M2 macrophages) (Chaintreuil et al., 2023). Using a protocol introduced in a previous work (Xu et al., 2019), we differentiated THP-1 cells to these three separate subtypes and tested their DNase activities in PCs (see Materials and methods). The three macrophage subtypes were incubated on 1.1 µm microbead-immobilized, SNS-coated surfaces for 30 min. The cell samples were then fixed, and F-actin was stained. The results revealed that all three macrophage subtypes displayed strong DNase activity in the PCs, as indicated by the SNS signals (Figure 2D). The SNS signal intensities are comparable among the three macrophage subtypes (Figure 2H). This result suggests that DNase activity in PCs is common among macrophage subtypes.

### Identification of the DNase in the PCs

After observing DNase activity in the PCs, we conducted a series of tests to identify the specific DNase type responsible for this activity. Although a soluble nuclease DNase II (Evans and Aguilera, 2003) is known to be recruited to phagosomes, it is unlikely responsible for DNase activity within PCs. This is because DNase II is acquired from lysosomes after the fusion of phagosomes and lysosomes, which occurs during the maturation stage of phagosomes, whereas DNase activity in PCs occurs during the PC formation stage. Additionally, DNase II is an acid nuclease (Varela-Ramirez et al., 2017) that functions in the low pH environment characteristic of mature phagosomes, a condition unavailable in unclosed PCs. Moreover, the SNS signal in the PCs is highly specific and confined to the microbeads, without degrading the SNS on the substrate regions near the microbeads. This suggests that DNase activity in the PCs is not mediated by soluble DNases, which would be expected to diffuse out of the PCs and be unable to concentrate the enzymatic activity on the microbeads.

We previously reported that a membrane-bound DNase, DNaseX (also known as DNase I-like 1), is recruited to podosomes of macrophages and invadopodia of cancer cells (Pal et al., 2021). Here, we hypothesize that the DNase in the PCs is also DNaseX. DNaseX is the only known membrane-bound DNase, which is anchored to the lipid membrane by a GPI linker (Shiokawa et al., 2007). We looked up the gene expression of DNaseX (DNASE1L1) and DNase I (DNASE1) in human hematopoietic cells on the website of Expression Atlas (Figure 3—figure supplement 1). The results revealed that macrophages and monocytes express the highest levels of DNASE1L1 mRNA among all hematopoietic cells, whereas DNASE1 mRNA levels in macrophages and monocytes are generally lower compared to other hematopoietic cells. The high expression level of DNaseX in macrophages suggests a potentially important, yet currently unknown, role of DNaseX in macrophage functions.

**Figure 3.**
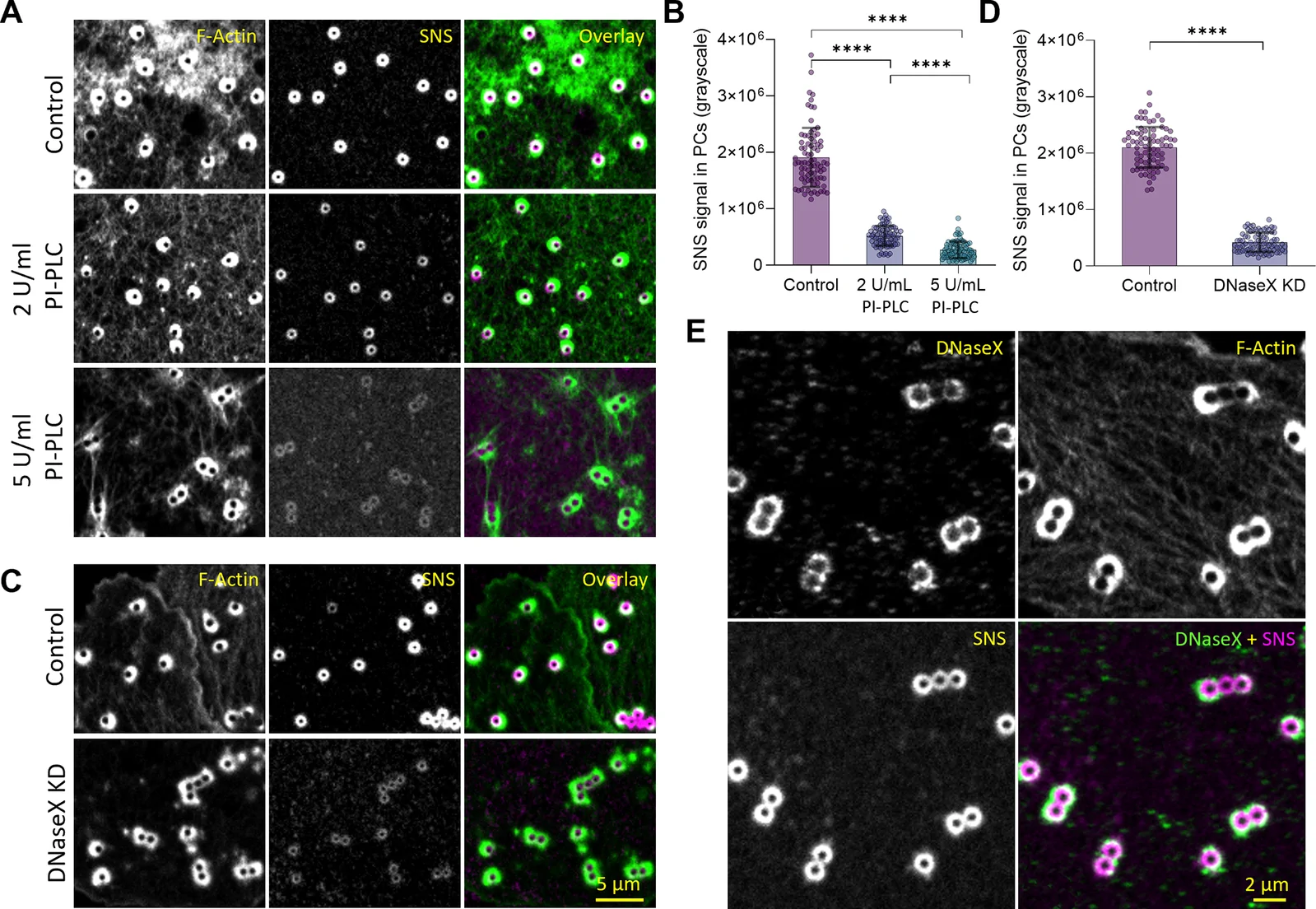
DNase in the phagocytic cups (PCs) was identified as membrane-bound DNaseX. (**A**) F-actin and surface-immobilized nuclease sensor (SNS) signals in the PCs of THP-1 macrophages treated with phosphoinositide phospholipase C (PI-PLC), which cleaves glycosylphosphatidylinositol (GPI) linkers of the putative membrane-bound DNase in the PCs. (**B**) PI-PLC treatment significantly reduced SNS signals in the PCs, indicating marked decreases in DNase activities. (****p<0.0001; Each data point represents one PC; n=3 experiments; error bars indicate SD.) (**C**) F-actin and SNS signals in the PCs of THP-1 macrophages with DNaseX knocked down by small interfering RNA (siRNA) interference. (**D**) DNaseX knockdown significantly reduced SNS signals in the PCs. (****p<0.0001; Each data point represents one PC; n=3 experiments; error bars indicate SD.) (**E**) Co-imaging of immunostained DNaseX, F-actin, and SNS signals in the PCs of THP-1 macrophages. Figure 3—source data 1.Numeric data and statistical analysis for the plots in Figure 3.

Because DNaseX is anchored to the cell membrane by a GPI linker, we first tested if the GPI linker is important for the DNase activity in the PCs. We treated the THP-1 cells with phosphoinositide phospholipase C (PI-PLC), a GPI-cleaving enzyme, and tested the cells on the SNS platform. The result showed that SNS signal intensity within PCs on microbeads significantly decreased upon the PI-PLC treatment, whereas F-actin structures of PCs remained intact (Figure 3A). When treated with PI-PLC at a concentration of 5 U/mL, the SNS signal intensity in the PCs was nearly reduced to the baseline level (Figure 3B). This result suggests that the DNase activity in the PCs is associated with a GPI-anchored nuclease, most likely DNaseX.

**Figure 3—figure supplement 1.**
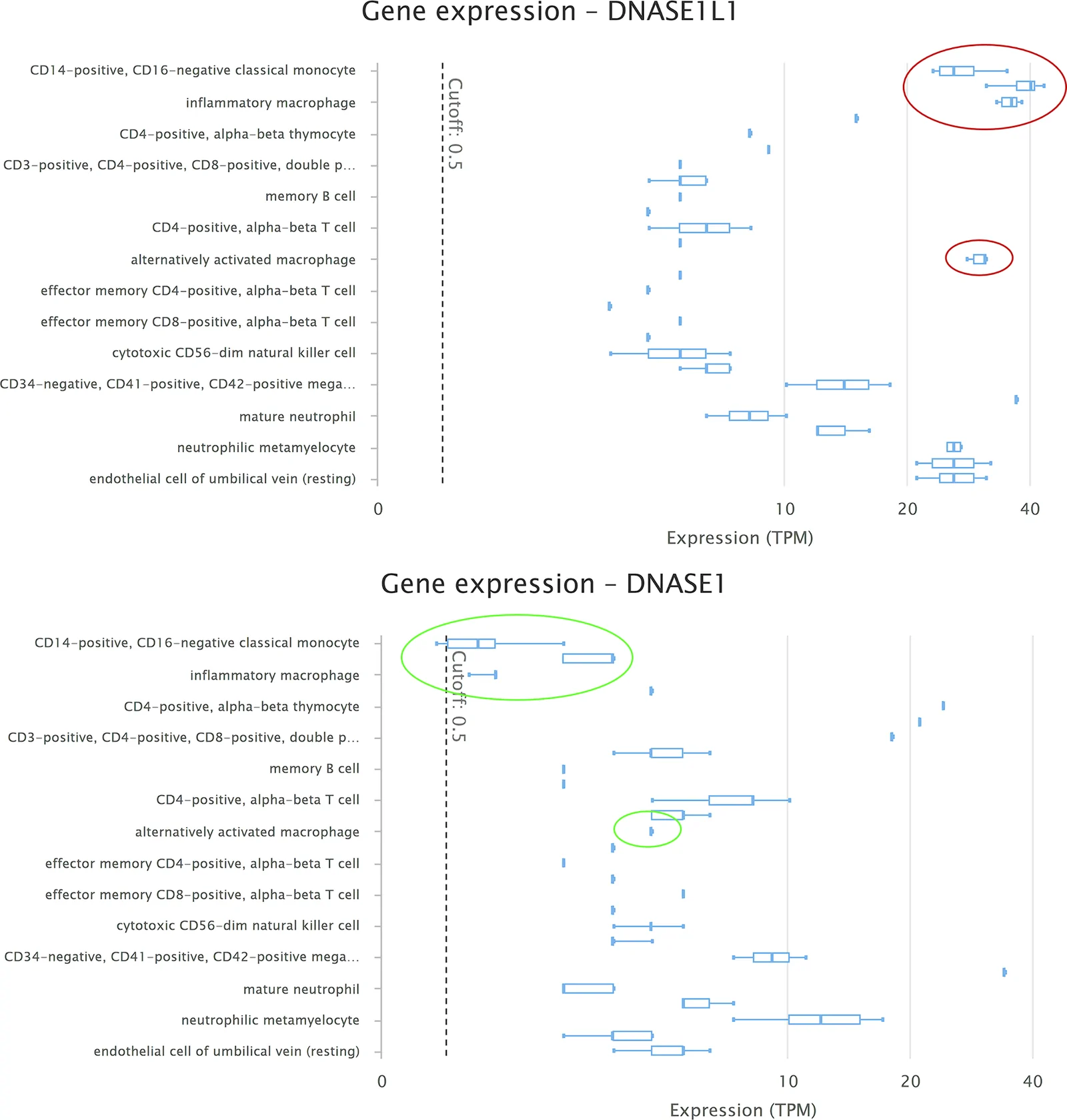
Gene expression of DNaseX (a.k.a. DNase I-like 1) in human hematological cells. The gene expression levels of DNaseX in human hematological cells are available in the Expression Atlas, a comprehensive database maintained by the European Bioinformatics Institute (EBI) that catalogs gene expression across various biological conditions and tissues. According to the data, mRNA of DNaseX is expressed at higher levels in macrophages and monocytes compared to other hematological cells, whereas mRNA of DNase I shows lower expression levels in macrophages relative to other hematological cell types.

**Figure 3—figure supplement 2.**
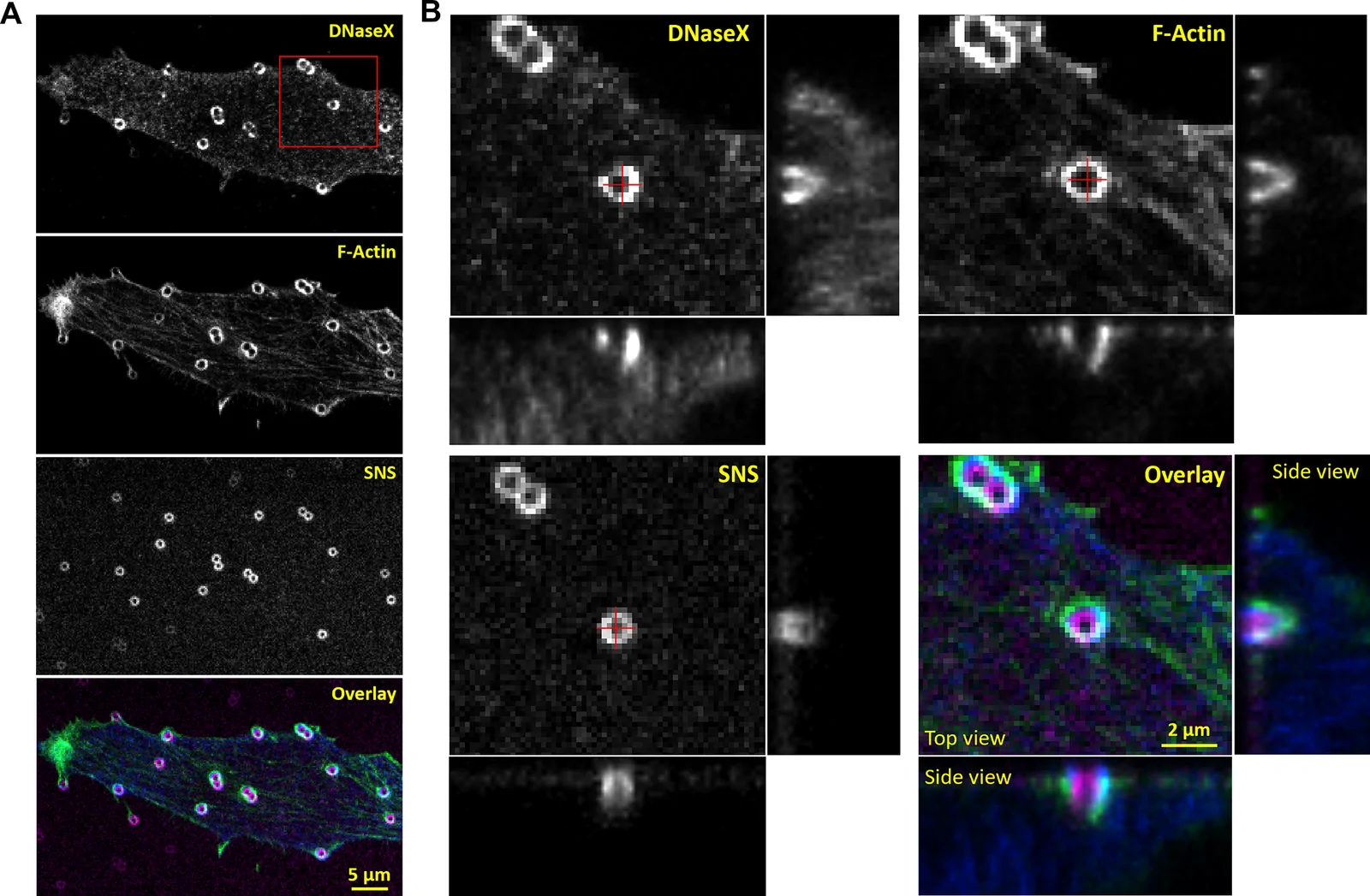
DNaseX was recruited onto microbeads beneath the domes of phagocytic cups (PCs). (**A**) Co-imaging of DNaseX (immunostained), F-actin, and surface-immobilized nuclease sensor (SNS) in a THP-1 macrophage. (**B**) 3D scan of DNaseX, F-actin, and SNS using confocal microscopy. All three signals are shown to be on the microbeads beneath the domes of the PCs, not on the substrate regions around microbeads.

Next, we utilized small interfering RNA (siRNA) to reduce DNaseX expression in cells. THP-1 macrophages were treated with siRNA targeting the mRNA of DNaseX, or non-targeting siRNA as a negative control, respectively. While THP-1 macrophages treated with non-targeting siRNA displayed normal SNS signals in the PCs, those treated with siRNA targeting DNaseX showed a significant reduction (p<0.0001) in SNS signal intensities in the PCs (Figure 3C and D). Meanwhile, the F-actin structures of the PCs were formed in both cases, showing no noticeable differences. This result further indicates that DNaseX is the primary DNase responsible for the observed DNA degradation in the PCs.

Finally, we conducted immunostaining using a DNaseX-specific antibody on THP-1 macrophages, which were detached by trypsin and cultured on an SNS-coated surface for 1 hr. Fluorescence imaging was performed to simultaneously visualize DNaseX, F-actin, and SNS signals. The result shows co-localization among these three signals around the microbeads (Figure 3E), confirming the presence of DNaseX in the PCs. 3D scan of DNaseX, F-actin, and SNS further shows that all these signals are on the microbeads beneath the domes of the PCs, not on the glass substrate regions around the microbeads (Figure 3—figure supplement 2). Based on these lines of evidence, we identified the DNase in the PCs of macrophages as GPI-anchored membrane-bound DNaseX.

### DNaseX is constitutively recruited to PCs without requiring the presence of DNA materials

**Figure 4.**
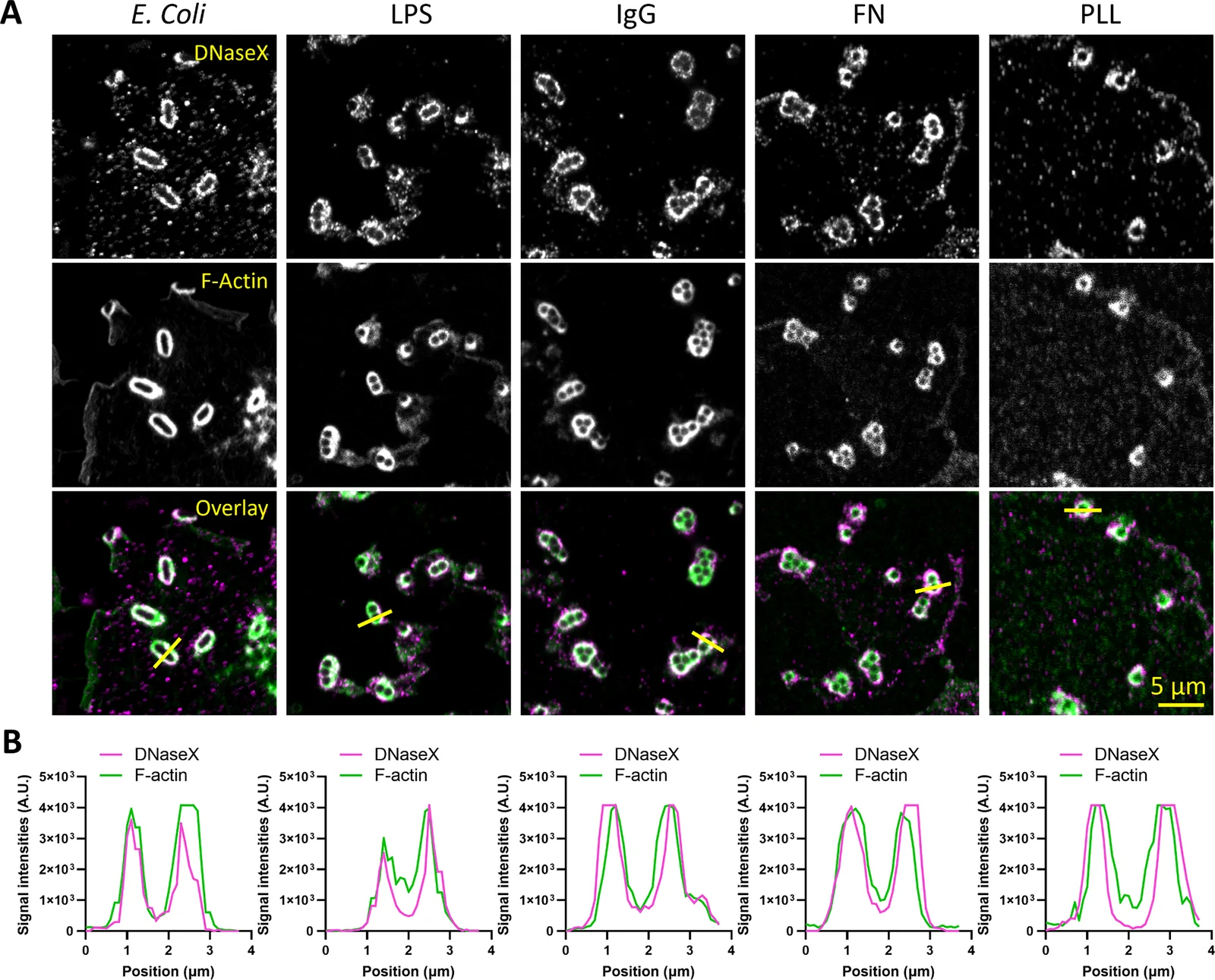
DNaseX is constitutively recruited to the phagocytic cups (PCs) without requiring the presence of DNA materials. (**A**) DNaseX in the PCs in response to surface-immobilized *E. coli*, or microbeads coated with various biomaterials, including lipopolysaccharide (LPS), immunoglobulin G (IgG), fibronectin (FN), and poly-L-lysine (PLL). DNaseX was immunostained with antibodies for imaging. (**B**) Line profiles show the co-localization of DNaseX and F-actin in the PCs on these five surfaces. Yellow lines in (**A**) mark the locations for the line profile analysis. Figure 4—source data 1.Numeric data for the plots in Figure 4.

We established that DNaseX is recruited to the PCs on SNS-coated microbeads. As SNS is essentially dsDNA, an immunogenic substance that could be sensed by DNA receptors such as Toll-like receptor 9 (Ashkar and Rosenthal, 2002) or cGAS (Sun et al., 2013), we asked whether the recruitment of DNaseX to PCs is specifically triggered by the presence of DNA on the microbeads. To answer this question, we prepared surfaces without SNS coating and instead decorated them with *E. coli* or microbeads coated with non-DNA biomolecules, including lipopolysaccharide (LPS), rabbit immunoglobulin G (IgG), fibronectin (FN), and poly-L-lysine (PLL). THP-1 macrophages were plated on these surfaces, and DNaseX immunostaining along with F-actin staining was performed (Figure 4A, Figure 4—figure supplement 1). Interestingly, the results showed that DNaseX and F-actin structures were present in the PCs on all particles, irrespective of the biomaterials in the PCs. The confocal images and respective line profile analysis results are shown in Figure 4A and B. This result suggests that DNaseX recruitment in the PCs is constitutive, without requiring the presence of DNA materials.

**Figure 4—figure supplement 1.**
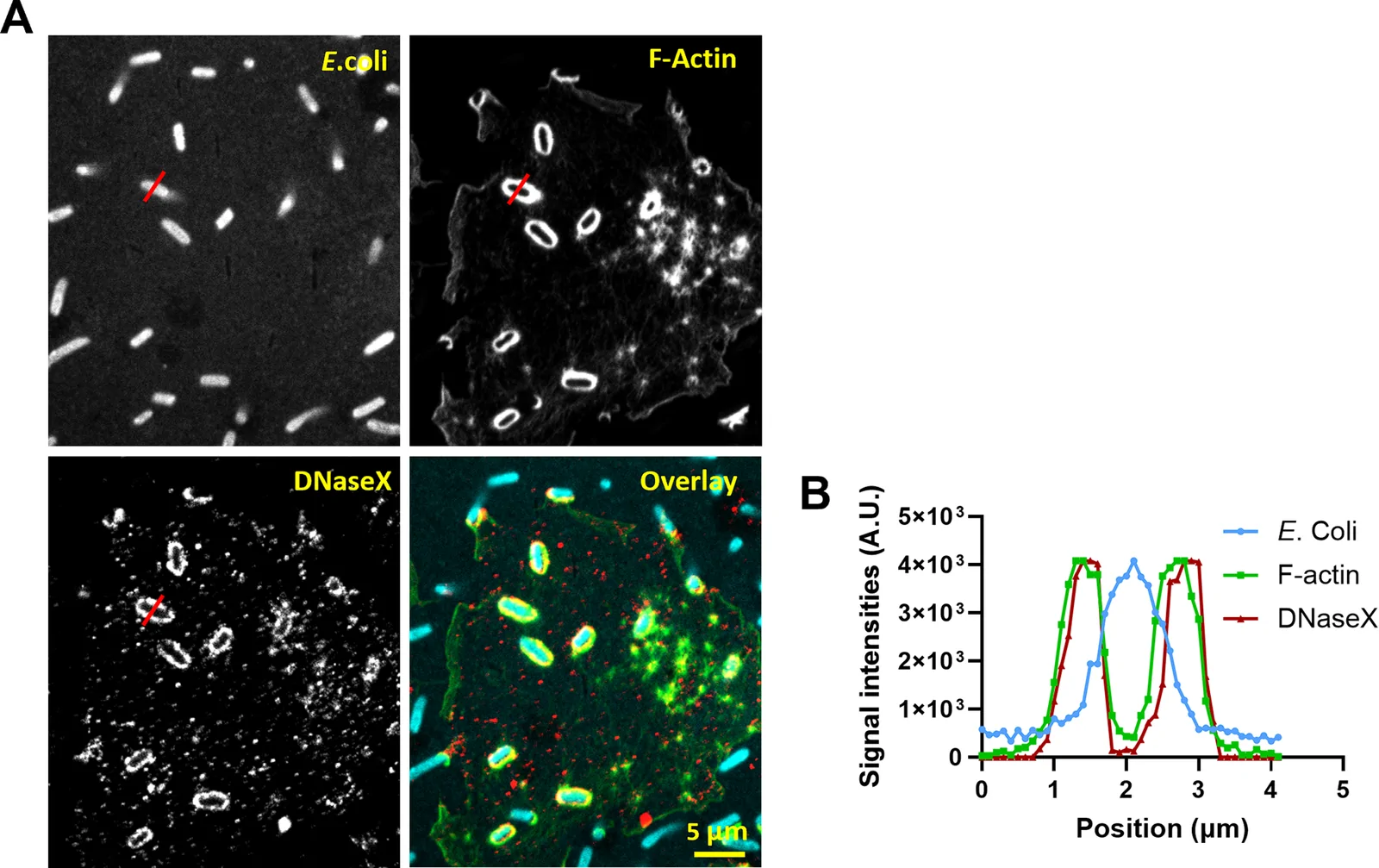
DNaseX was recruited to the phagocytic cups (PCs) in response to surface-immobilized *E. coli*. (**A**) Co-imaging of *E. coli*, F-actin, and immunostained DNaseX underneath a THP-1 macrophage. (**B**) Line profiles of *E. coli*, F-actin, and immunostained DNaseX. The region of analysis is marked with a red line in (**A**).

**Figure 4—figure supplement 2.**
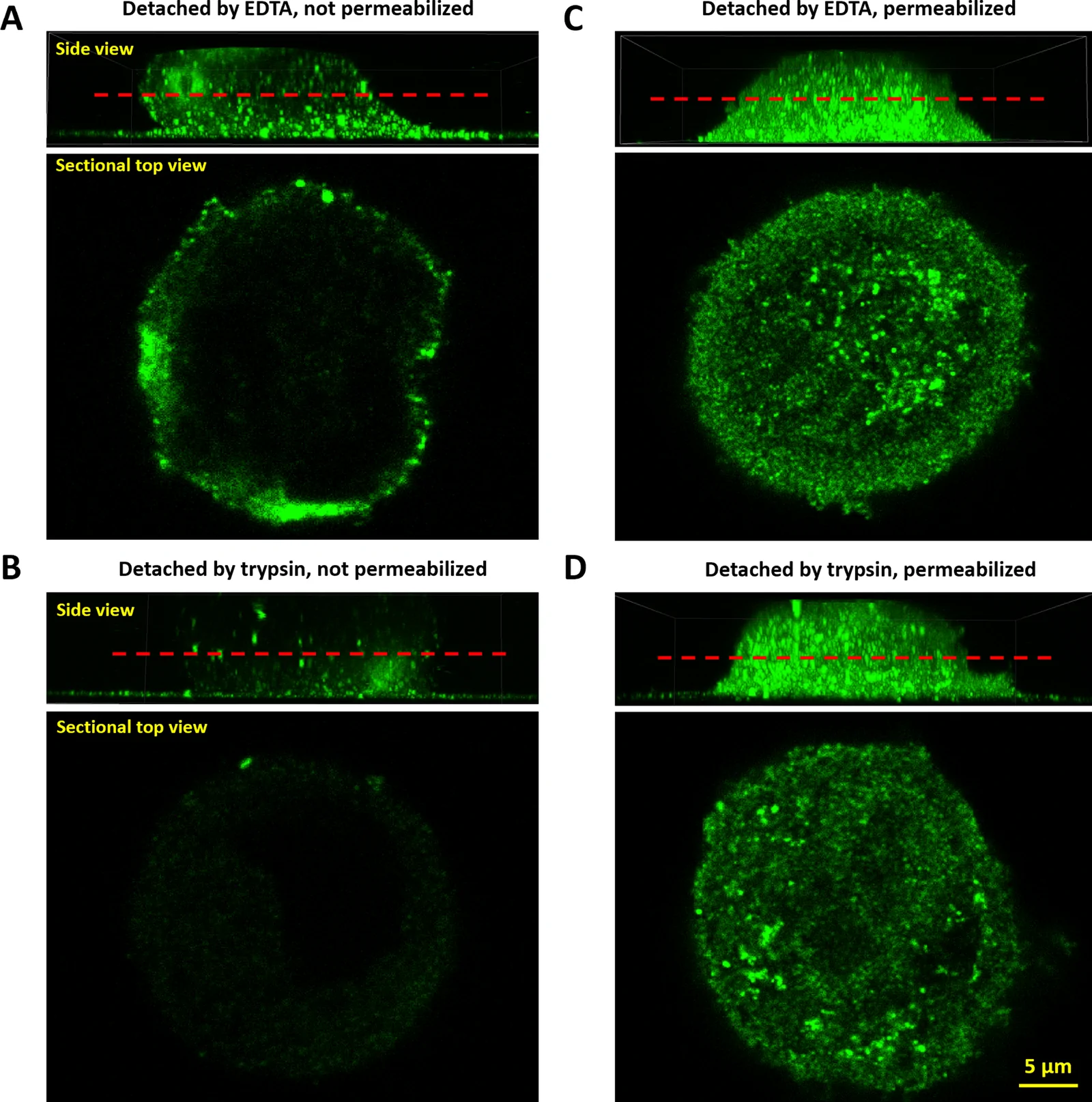
DNaseX is found in both plasma membrane and inside cells. (**A**) Confocal scan of DNaseX on the plasma membrane of macrophages, which were detached by ethylenediaminetetraacetic acid (EDTA) and not permeabilized during cell fixation. The red dashed line indicates the section of 3D imaging displayed in the top view. (**B**) Confocal scan of DNaseX on the plasma membrane of macrophages, which were detached by trypsin and not permeabilized during cell fixation. (**C**) Confocal scan of DNaseX on the plasma membrane and in the cytoplasm of macrophages, which were detached by EDTA and permeabilized during cell fixation. (**D**) Confocal scan of DNaseX on the plasma membrane and in the cytoplasm of macrophages, which were detached by trypsin and permeabilized during cell fixation.

**Figure 4—figure supplement 3.**
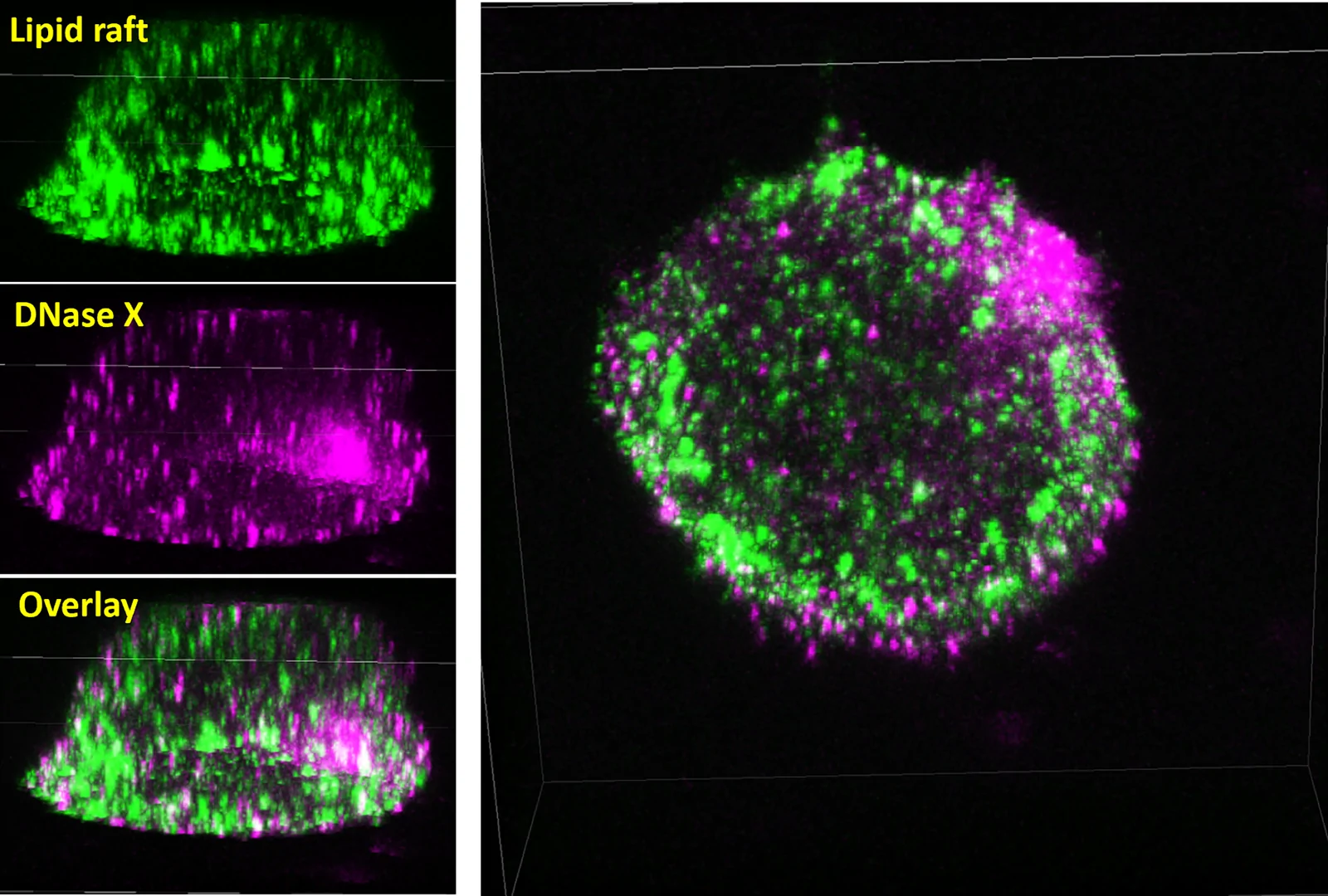
No co-localization between DNaseX and lipid rafts on the cell membrane of a macrophage. Confocal scanning reconstructed the three-dimensional distribution of lipid rafts and DNaseX clusters on the plasma membrane of a macrophage. No apparent co-localization was observed between these two signals. The lipid rafts were stained with a lipid raft labeling kit (V-34405, Molecular Probes). Related to Video 2.

### DNaseX in PCs is recruited intracellularly, not from the plasma membrane

As GPI-anchored proteins, DNaseX is synthesized in the endoplasmic reticulum, processed in the Golgi apparatus, and transported to the plasma membrane, likely via secretory vesicles. Our results showed that DNase activity is generally specific to the PCs. Here, we investigated the DNaseX distribution on the plasma membrane and in the cytoplasm. Because fusion with fluorescent proteins could interfere with DNaseX function by disrupting GPI anchor attachment at the C-terminus or enzymatic activity at the N-terminus, we primarily relied on immunostaining to determine the cellular localization of DNaseX.

We performed immunostaining combined with confocal 3D imaging to examine the subcellular localization of DNaseX in adherent macrophages. To assess membrane association, cells were detached using either ethylenediaminetetraacetic acid (EDTA) or trypsin, which preserve or remove DNaseX from the plasma membrane, respectively. EDTA provides gentle detachment that maintains membrane proteins, whereas trypsin, a protease, degrades membrane-bound proteins during cell detachment. During immunostaining, the cell membrane was selectively permeabilized to visualize either total cellular DNaseX or only the membrane-bound fraction. As shown in Figure 4—figure supplement 2, DNaseX was predominantly detected in submicron-sized plaques on the plasma membrane and in punctate intracellular clusters, likely corresponding to vesicles. DNaseX plaques are not associated with lipid rafts (Figure 4—figure supplement 3 and Video 2), despite previous reports that GPI-anchored proteins are typically enriched in these membrane domains (Wang et al., 2013).

**Video 2.** 3D reconstruction of DNaseX and lipid raft imaged in a THP macrophage.

**Figure 5.**
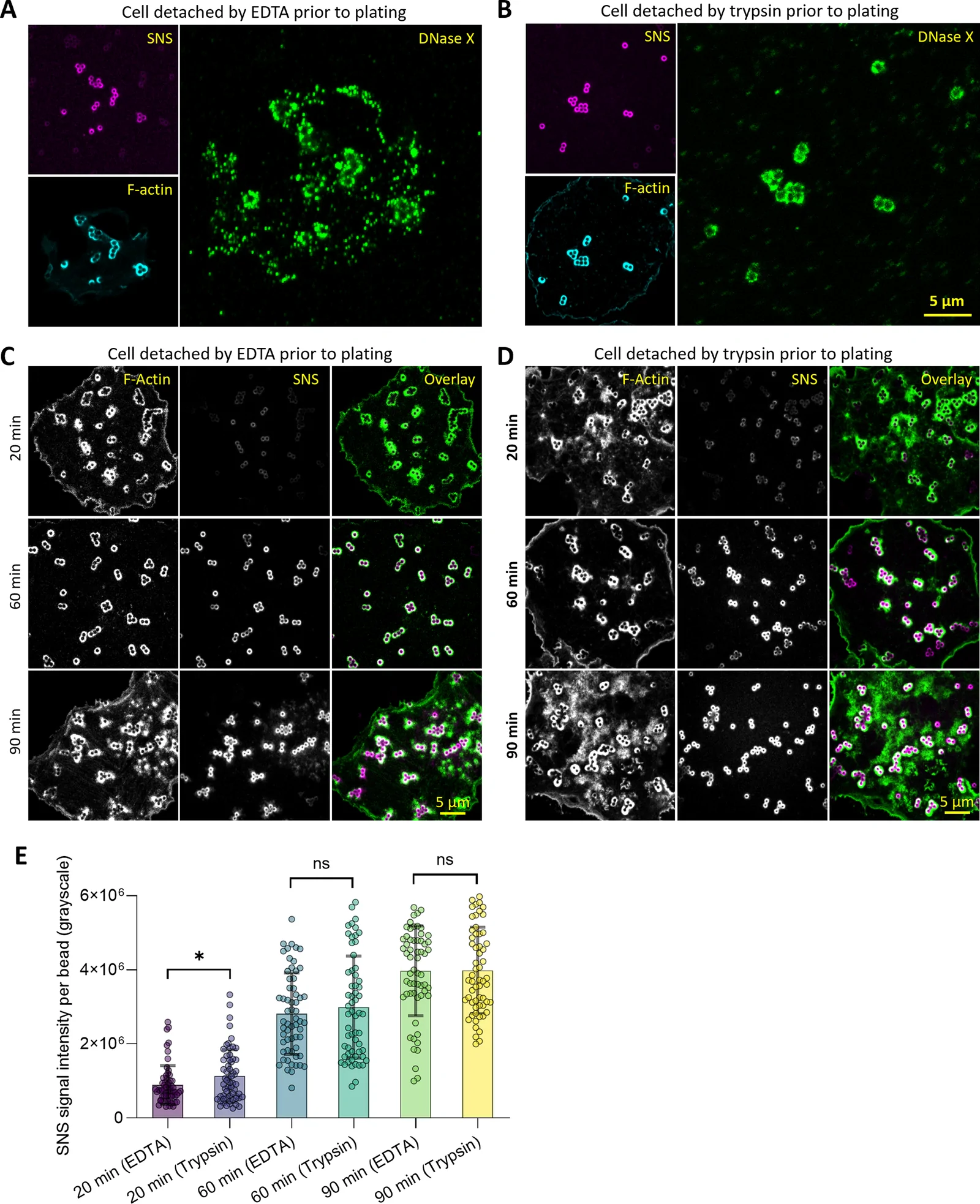
Distribution of DNaseX in the phagocytic cups (PCs), on the plasma membrane and inside the cell. (**A, B**) Surface-immobilized nuclease sensor (SNS) signal, F-actin, and immunostained DNaseX on the ventral surfaces of adherent THP-1 macrophages, which were detached from a culture flask by either ethylenediaminetetraacetic acid (EDTA) (**A**) or trypsin (**B**) prior to cell plating. The cells were incubated on the SNS surfaces for 1 hr. (**C, D**) DNase activities in the PCs indicated by SNS signals with 20, 60, and 90 min incubation times, respectively. The macrophages were detached by either EDTA (**C**) or trypsin (**D**). (**E**) SNS signal intensities in PCs. (*p<0.05; ns p>0.05; Each data point represents one PC; n=3 experiments; error bars indicate SD.) Figure 5—source data 1.Numeric data for the plots in Figure 5.

Since DNaseX is present in both the plasma membrane and cytoplasm, we investigated which region contributes DNaseX to PCs during phagocytosis. To do this, we treated THP-1 macrophages with either EDTA or trypsin, to preserve or remove DNaseX on the plasma membrane, respectively. These cells were then plated on SNS surfaces to observe DNaseX recruitment to the PCs and the corresponding DNase activities. As shown in Figure 5A, when the cells were treated with EDTA, DNaseX existed in both the PCs and submicron-sized plaques, which are not associated with phagocytosis. In contrast, when the cells were detached by trypsin, DNaseX was only present at the PCs (Figure 5B). DNase activities in PCs indicated by SNS signals were normal regardless of cell detachment methods, suggesting that DNaseX in the PCs is not derived from pre-existing membrane clusters but is instead recruited from an intracellular pool of DNaseX within macrophages.

We further compared the time-dependent SNS signal intensities in PCs between EDTA-detached and trypsin-detached macrophages (Figure 5C and D). As shown in Figure 5E, the differences in DNase activity of PCs between these two conditions were marginal or insignificant at 20, 60, and 90 min of cell incubation. This suggests that eliminating DNaseX in the cell membrane by trypsin does not affect DNase activity in the PCs, supporting the idea that DNaseX in the PCs is recruited from the cytoplasm rather than the plasma membrane.

### Actin polymerization is correlated with DNase activity in PCs, but not with DNaseX recruitment

We have consistently observed co-localization of F-actin and DNaseX in PCs. This observation prompted us to consider the role of F-actin in the DNase activity of DNaseX. Since membrane-bound DNaseX requires direct physical contact with solid DNA materials for degradation, we speculated that F-actin polymerization exerts pressure on the plasma membrane within the PCs, bringing membrane-bound DNaseX into close proximity with solid DNA materials and thereby facilitating the degradation.

**Figure 6.**
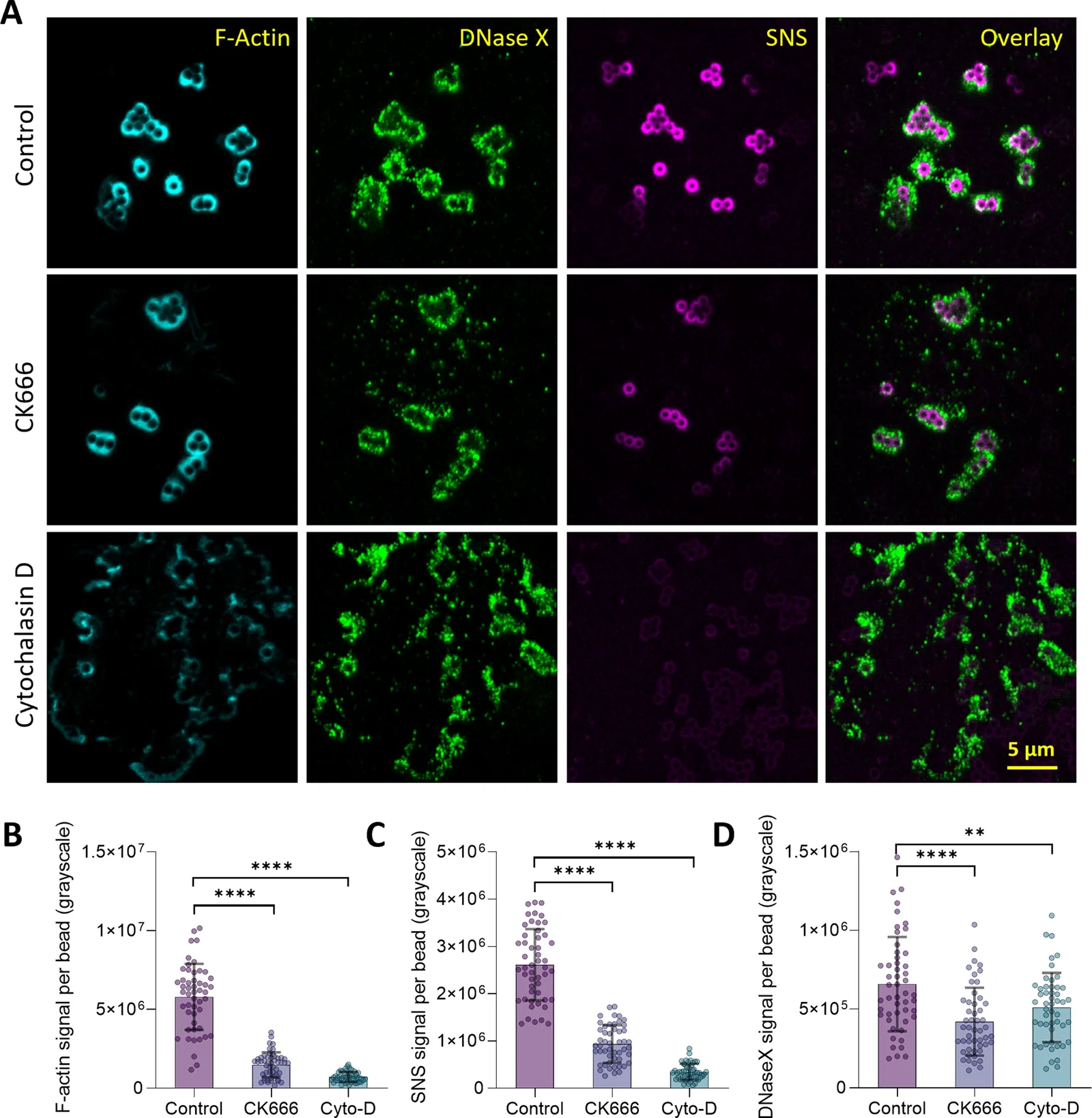
F-actin structure is correlated with DNase activity but not with DNaseX recruitment in the phagocytic cups (PCs). (**A**) Images of F-actin, DNaseX, and surface-immobilized nuclease sensor (SNS) signals in the PCs with the macrophages treated with dimethyl sulfoxide (DMSO) (control), 100 µM CK666, or 1 µM cytochalasin D, respectively. (**B–D**) Signal intensities of F-actin, SNS, and DNaseX signals in the PCs with the above treatments. (****p<0.0001; **p<0.01; Each data point represents one PC; n=3 experiments; error bars indicate SD). Figure 6—source data 1.Numeric data and statistical analysis for the plots in Figure 6.

To test this hypothesis, we disrupted actin polymerization using 100 µM CK666 or 1 µM cytochalasin D. CK666 inhibits the Arp2/3 complex, thereby preventing actin branching, while cytochalasin D caps the barbed (plus) ends of F-actin filaments, blocking their elongation. We imaged F-actin, DNaseX, and SNS signals in macrophages treated with these two reagents or dimethyl sulfoxide as control (Figure 6A). Both reagents effectively reduced F-actin and SNS signals in the PCs (Figure 6B and C) while DNaseX levels in the PCs were not significantly affected (Figure 6D). These results indicate that actin polymerization in the PCs is not correlated with DNaseX recruitment, but correlated with its enzymatic activity within the PCs.

This observation supports the hypothesis that F-actin polymerization generates the protrusive force to push membrane-bound DNaseX to DNA materials for the latter’s degradation. It can also explain why DNaseX in plaques on the plasma membrane generally does not show DNase activity on the SNS-coated surface. This could be due to that DNaseX, as membrane-bound DNase, requires direct physical contact with solid DNA materials on the surface to initiate catalytic degradation, but there is typically a narrow gap, often hundreds of nanometers, between the cell membrane and the underlying substrate (Dai et al., 2021), which effectively blocks the degradative activity of DNaseX plaques.

### Macrophages intensively degrade eDNA in bacterial biofilm by physical contact

A preliminary study was conducted to investigate how macrophages degrade eDNA in bacterial biofilms. *S. aureus* is the leading cause of healthcare-associated infections and poses a significant medical and economic burden on society (Li et al., 2023). These infections are difficult to cure because biofilms formed by *S. aureus* enhance their resistance to antibiotic treatments (Peng et al., 2022; Lee et al., 2020), and eDNA is a major structural component of the biofilms (Campoccia et al., 2021; Okshevsky et al., 2015).

**Figure 7.**
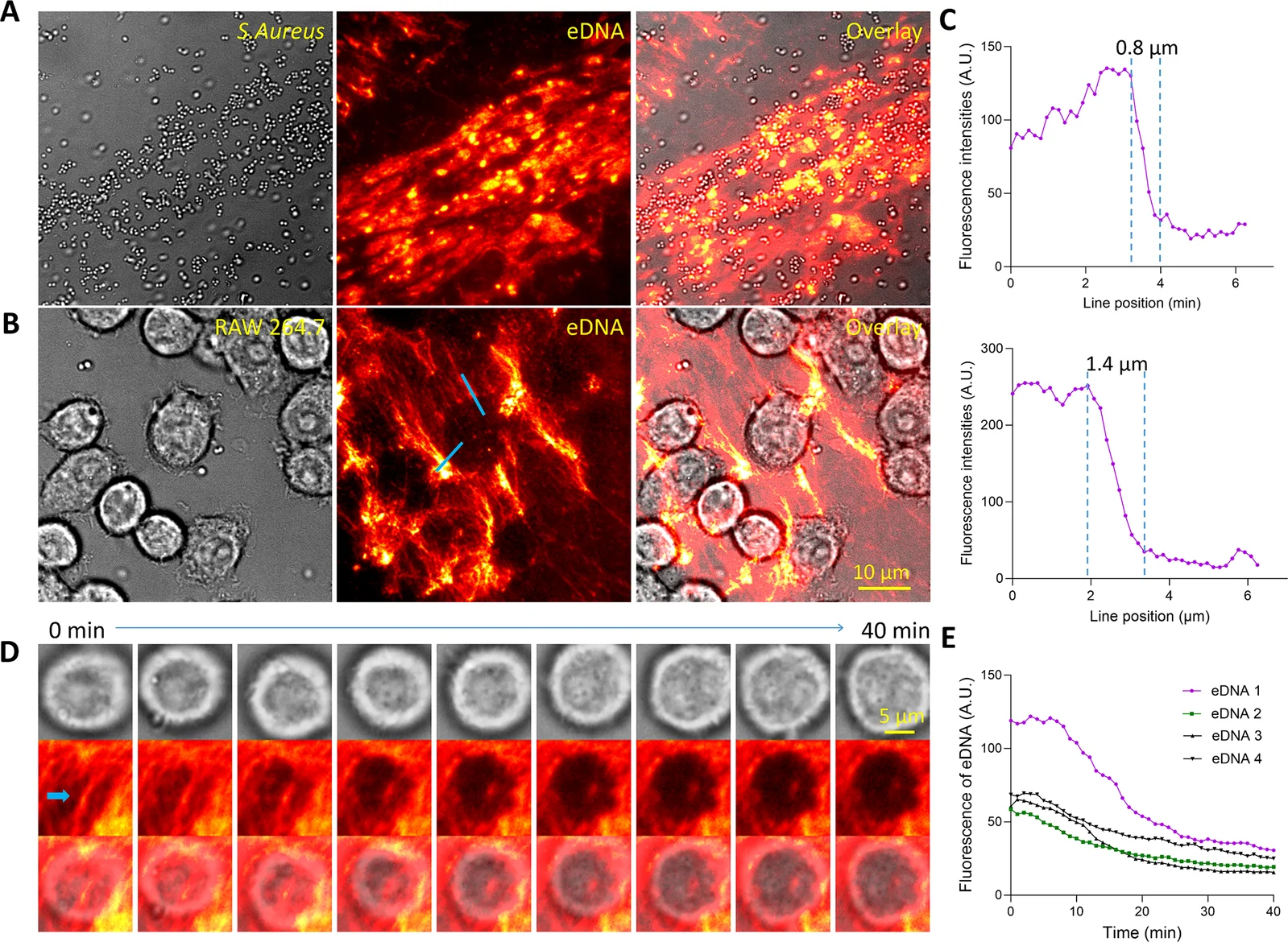
Macrophages degrade extracellular DNA (eDNA) in biofilms by physical contact. (**A**) eDNA structures in *S. aureus* biofilms. The eDNA was stained with DITO-1. (**B**) RAW macrophages incubated on *S. aureus* biofilms for 30 min. The filamentous eDNA structures were degraded under cell bodies. (**C**) Line profile analysis of eDNA degradation regions indicated by blue lines in (**B**), which are shown to have sharp boundaries with ~1 µm transition from undegraded region to degraded region, suggesting that the eDNA degradation was mediated by non-diffusive DNase. (**D**) Time-series images of eDNA degradation by a RAW macrophage. Related to Video 3. The eDNA filament (indicated by a blue arrow) became gradually shortened during the degradation. (**E**) Analysis of eDNA degradation by macrophages. The degradation process typically spans 20–30 min. Figure 7—source data 1.Numeric data for the plots in Figure 7.

We prepared biofilms formed by *S. aureus*. As shown in Figure 7A, DNA staining indeed revealed filamentous eDNA structures within *S. aureus* colonies cultured on a Petri dish for 2–3 days. By a gentle rinse, the bacteria were washed off from the biofilm; RAW macrophages were then plated on the surface and incubated for 30 min. Cells were then fixed for imaging. Figure 7B shows that eDNA in biofilms was degraded in a pattern highly co-localized with macrophage cell bodies. The boundaries of the eDNA-degraded regions are sharp, with a one micron-scale transition from non-degraded to degraded areas (Figure 7C), suggesting that RAW macrophages degraded eDNA through direct physical contact rather than via diffusive soluble DNases. Live-cell imaging in Figure 7D and Video 3 further confirms that macrophages degrade eDNA structures within the biofilm, as filamentous eDNAs were progressively severed and dissolved beneath the cell body. A macrophage typically takes 20–30 min to degrade eDNA beneath its cell body (Figure 7E), demonstrating high efficiency in localized eDNA dissolution in bacterial biofilms.

**Video 3.** Macrophages degrading extracellular DNA (eDNA) structure in bacterial biofilm. The biofilm was formed by *S. aureus*. Cells are RAW macrophages. The actual duration of cell activities in the video is 1 hr.

While the results showed that macrophages degrade eDNA in biofilms through physical contact, it has not been confirmed that this degradation is mediated by DNaseX within the PC, as eDNA filaments are not expected to induce the formation of a typical PC structure. An alternative possibility is that DNaseX localized on the cell membrane, including within the plaque-like clusters (Figure 4—figure supplement 2), comes into direct contact with eDNA and mediates its degradation through physical contact.

## Discussion

Phagocytosis is a crucial process for eliminating pathogens and clearing dead self-cells, playing a vital role in immune defense and homeostasis. Conventionally, PC formation and enzymatic degradation have been viewed as separate stages of phagocytosis. Our findings challenge this view by showing that macrophages recruit membrane-bound DNaseX to PCs at an unexpectedly early stage, coinciding with PC formation. Recruitment of DNaseX to PCs was observed across all macrophage types tested, in response to both pathogens and microbeads coated with various biomaterials. These findings indicate that DNase activity is a constitutive component of PC formation rather than a downstream event. The early and ubiquitous presence of DNaseX in the PCs likely reflects an evolutionarily conserved strategy in macrophages for immune defense. Some pathogens such as *Legionella pneumophila* have evolved mechanisms to evade lysosomal hydrolases by disrupting phagosome maturation (Amer et al., 2006). By degrading potential eDNA during the PC formation, DNaseX may provide a preemptive assault against pathogenic genetic material, reducing the risk of virulence gene transfer or pathogen-mediated hijacking of host pathways.

In addition to targeting individual micron-sized particles, DNaseX in the PCs may also allow macrophages to efficiently degrade bulky eDNA structures. Both pathogens and decomposing host self-cells release eDNA. Many bacteria integrate eDNA as a major structural material to form bacterial biofilms, which shield bacteria from immune attack of the hosts and antibiotic treatment (Jamal et al., 2018). On the other hand, in response to microbial cues and endogenous cytokines, neutrophils can release NETs (Papayannopoulos, 2018; Kaplan and Radic, 2012), which are composed of chromatin, to ensnare pathogens and fight infections (Papayannopoulos, 2018). However, excessive accumulation of NETs in organs such as the brain, lungs, heart, and kidneys can cause significant organ injuries (Cahilog et al., 2020; Czaikoski et al., 2016), requiring a timely clearance by macrophages. Although soluble DNases such as DNase I (Napirei et al., 2004), DNase II (Kawane et al., 2006), and Caspase-activated DNase (Nagata, 2005) have been implicated in DNA clearance, these enzymes have limitations to degrade bulky eDNA structures as soluble DNases can easily be diluted by body fluid and diffuse away from target eDNA. Our discovery of DNaseX in PCs, along with direct imaging of macrophages degrading eDNA in biofilms through physical contact, demonstrates that macrophages utilize membrane-bound DNase to degrade bulky eDNA structures. This process either directly degrades the eDNA structures or processes them into smaller fragments suitable for phagocytic uptake. The discovery of membrane-bound DNaseX in PCs provides new insight into how macrophages clear bulky eDNA released by pathogens or damaged self-cells.

Together, the identification of DNaseX in PCs highlights a previously unrecognized mechanism of innate immunity mediated by macrophages. This early, membrane-bound DNase activity equips macrophages with a rapid and spatially focused strategy to neutralize pathogenic or immunogenic eDNA. This discovery opens potential avenues for therapeutic development, such as enhancing DNaseX activity in macrophages to combat biofilm-induced chronic infections and to remove self-DNA that drives inflammation or autoimmune disorders. One limitation of the current study is that the upstream signals responsible for DNaseX recruitment to PCs have not yet been identified. Future work will be needed to define the trafficking pathways of DNaseX recruitment to PCs, and whether its activity can be leveraged to enhance host defense or mitigate DNA-driven inflammatory pathologies.

## Materials and methods

### Synthesis of SNS

SNS is a dsDNA decorated with a dye, a quencher, and a biotin, reporting DNase activity by fluorescence gain. The other two DNase sensors are dye-labeled dsDNA or ssDNA, reporting DNase activity by fluorescence loss. The DNA strands were customized and purchased from Integrated DNA Technologies with sequences and modifications as given in Table 1.

**Table 1.** Oligonucleotide sequences for the synthesis of DNase sensors that report DNase recruitment in the macrophage phagocytic cups.

DNA strands for DNase sensor	Oligonucleotide sequences
Upper DNA strand for SNS	5′-GGGCGGCGACCTCAGCAT/3BHQ_2/3′
Lower DNA strand for SNS	5′-/5BiosG/T/iAtto647N/ATGCTGAGGTCGCCGCCC-3′/
Upper strand for dye-labeled dsDNA	5′-GGGCGGCGACCTCAGCAT-3′
Lower strand for dye-labeled dsDNA	5′-/5BiosG/ATGCTGAGGTCGCCGCCC/Atto647N/-3′
Dye-labeled ssDNA	5′-/5BiosG/ATGCTGAGGTCGCCGCCC/Atto647N/-3′

To prepare dsDNA-based DNase sensors, the respective DNA upper and lower strands were mixed at a molar ratio of 1.1:1.0 and annealed at 90°C at a final concentration of 10 µM. DNase sensors were aliquoted and stored at 4°C for regular use and –20°C for long-term storage.

### Immobilization of polystyrene beads on glass-bottom Petri dishes

To study DNase recruitment in macrophage PCs, polystyrene microbeads were permanently immobilized on a glass-bottom Petri dish, serving as the platform for macrophage incubation and imaging. First, a 50 µg/mL PLL (P4707, Sigma-Aldrich) in Milli-Q water was incubated on the glass-bottom Petri dish (D-35-14-1.5-N, Cellvis) for 30 min at room temperature, followed by three washes with Milli-Q water. Subsequently, polystyrene beads (LB3, LB11, or LB30, Sigma-Aldrich) or fluorescent beads (B0100, Thermo Fisher Scientific) were mixed with Milli-Q water at a 1:1000 (vol/vol) ratio and vortexed for 2.5 min. This bead suspension was incubated on the glass-bottom Petri dish for 30 min at room temperature. The microbeads should be adsorbed on the glass surface by the positively charged PLL coating.

After incubation, the bead solution was carefully drawn by a pipette to minimize liquid residue on the dish. The Petri dish was then baked on a hot plate at 120°C for 1 min to ensure permanent immobilization of the beads. Following this, the dish was allowed to cool for a few minutes at room temperature and then washed thoroughly with Milli-Q water.

### Coating SNS on the bead-immobilized glass surface

To report DNase activity in PCs, the microbead-immobilized glass surface was further coated with the DNase sensor SNS using biotin-avidin interaction. First, a mixture solution of 100 µg/mL BSA-biotin (biotin-conjugated BSA; 29130, Thermo Scientific) and 5 µg/mL FN (1918-FN, R&D Systems) in phosphate-buffered saline (PBS, 46-013 CM, Corning) was incubated on the polystyrene bead-immobilized glass-bottom Petri dish for 15 min at 4°C. Both BSA-biotin and FN were physically adsorbed on the glass surface. FN was intended to enhance cell adhesion on the surface, and BSA-biotin provides the biotin tag for avidin immobilization, facilitating the subsequent attachment of biotin-tagged SNS.

After incubation, the surface was thoroughly rinsed three times with cold PBS. Next, a solution of 50 µg/mL neutravidin (31000; Thermo Scientific) in PBS was incubated on the Petri dish for 15 min at 4°C, followed by three washes with cold PBS. Finally, the bead-immobilized surface was incubated with a solution of 0.1 µM SNS in PBS for 15 min at 4°C and washed three times with cold PBS. At this stage, the bead-immobilized and SNS-coated glass surface was ready for cell plating and further experiments.

### Coating plasmid DNA on the bead-immobilized glass surfaces

50 μg/mL PLL in water was added to the well of a microbead-decorated glass-bottom Petri dish and incubated for 20 min. The Petri dish was washed with water three times. Plasmid DNA was extracted from *E. coli* transformed with LifeAct-GFP using a high-speed plasmid mini kit (IB47101, IBI Scientific). The immobilization of plasmid DNA is enabled by the electrostatic force as the PLL coating is positively charged and the DNA backbone is negatively charged. A 50 µg/mL plasmid DNA solution was incubated on a PLL-coated and bead-decorated glass surface for 30 min at room temperature, and the glass surface was washed with PBS three times. The DNA was stained with 1 µM SYTOX Green and washed with PBS three times. RAW macrophages were plated in the Petri dish and incubated for 1 hr in an incubator (5% CO_2_ and 37°C). The sample was imaged directly without cell fixation.

### Coating other biomaterials on the bead-immobilized glass surfaces

To test what biomaterials may elicit the recruitment of DNaseX to PCs, biomaterials other than DNA materials were coated on bead-immobilized glass-bottom Petri dishes. These biomaterials include: 20 μg/mL LPS-EB-Biotin (tlrl-lpsbiot, Invivogen), 20 μg/mL rabbit IgG-Biotin (011-0602, Rockland), 10 μg/mL FN (37582, Thermo Scientific) in PBS, and 50 µg/mL PLL (P4707, Sigma-Aldrich). The biotin-labeled LPS and IgG were immobilized on the surfaces which were pre-coated with BSA-biotin and neutravidin. FN and PLL were directly coated on the bead-decorated glass Petri dishes through physical adsorption with 1 hr incubation at 4°C. These surfaces were washed with cold PBS three times and were ready for cell plating.

### Immobilization of *E. coli* on SNS surfaces

In addition to polystyrene beads, we used *E. coli* (DH5α) as pathogen particles in the phagocytosis study. To immobilize *E. coli*, a glass-bottom Petri dish was coated with 50 µg/mL PLL in Milli-Q water for 30 min at room temperature, followed by three washes with Milli-Q water. The DH5α strain *E. coli* (18265017, Thermo Fisher Scientific) or fluorescently labeled *E. coli* (601371, Cayman Chemical) were prepared in PBS and transferred onto the PLL-coated Petri dish, where *E. coli* was incubated for 30 min at room temperature. To immobilize the *E. coli* on the PLL-coated surface, 0.5% glutaraldehyde (G6257, Sigma-Aldrich) was added and incubated for 15 min. Afterward, the surface was washed three times with Milli-Q water and treated with 10 mg/mL sodium borohydride (200050250, Thermo Scientific) to quench any autofluorescence potentially arising from glutaraldehyde. Finally, the surface was washed three times with Milli-Q water and was ready for macrophage plating.

### Human macrophages derived from PBMCs

Human macrophages were acquired by extracting and differentiating monocytes from blood samples. These samples were sourced from leukocyte reduction system (LRS) chambers, a cone-shaped disposable device used in blood transfusion medicine to eliminate white blood cells (leukocytes) from blood products. Consequently, the used LRS chambers contain an enriched level of leukocytes, making them a valuable source for leukocyte extraction.

One used LRS chamber was collected in Hoxworth blood center right after the blood donation (Figure 2—figure supplement 1A). Within 1 hr after the chamber collection, a 5 mL blood sample (citrate dextrose solution A was used as the anticoagulant during blood donation) was drained out from the chamber to a 50 mL centrifuge tube. A 15 mL PBS buffer (PBS without divalent ions such as Ca^2+^ or Mg^2+^) supplemented with 2 mM EDTA and 2% fetal bovine serum (FBS, 30-2020, ATCC) was added to the blood sample and gently mixed by rotating the tube.

Add 3 mL of density gradient medium (Lymphoprep, Catalog #07801, STEMCELL Technologies) to a fresh 15 mL tube. Carefully add 6 mL prepared blood sample on top of the gradient medium. Ensure that a clear separating line forms between the gradient medium and the blood sample. Centrifuge the sample at 400×*g* for 15 min using a swing-bucket centrifuge with the brake OFF.

Four layers of substances should be formed in the tube (Figure 2—figure supplement 1B). From top to bottom: The top layer consists of yellow-colored plasma. Below that is a thin and non-transparent layer of peripheral blood mononuclear cells (PBMCs). The third layer is the gradient medium. The bottom layer is red blood cells. Carefully harvest the PBMCs by inserting the pipette directly through the upper plasma layer to the second layer where PBMCs are located. Alternatively, the plasma layer can be removed and then collect the PBMCs. The cells are suspended in 2 mL PBS buffer (2 mM EDTA and 2% FBS). PBMCs are now ready for cryogenic storage or monocyte extraction.

### Monocyte extraction from PBMCs

A monocyte isolation kit (Catalog #19669, STEMCELL Technologies) was utilized to extract monocytes via negative selection (Figure 2—figure supplement 1C). This kit employs paramagnetic beads coated with antibodies that bind to all blood cells except monocytes. Cells captured by the beads are removed from the samples, leaving behind the monocytes. Refer to the detailed protocol provided by the manufacturer of the monocyte isolation kit. The isolated monocytes are cultured in RPMI-1640 medium (30-2001, ATCC) supplemented with 10% FBS and 1× penicillin-streptomycin.

### Differentiate monocytes into macrophages

The monocytes were differentiated into macrophages following established protocols: Human monocytes were cultured in a medium supplemented with 50 ng/mL macrophage colony-stimulating factor (M-CSF, 216-MCC, R&D Systems) for 4 days and cultured in a medium supplemented with 100 ng/mL M-CSF for another 4 days. The differentiated monocytes became adherent and were harvested using an EDTA-based mild cell-detaching reagent.

### Cell culture

Macrophage cell lines (RAW264.7 and THP-1) and monocyte-derived human macrophages were cultured and adopted for the study. RAW264.7 and human monocytes were cultured in Dulbecco’s Modified Eagle Medium (DMEM; 11995-065, Gibco) supplemented with 10% FBS albumin (35-011-CV, Corning) and 1% penicillin/streptomycin (30-002 CI; Corning). THP-1 cells were cultured in RPMI-1640 medium (112-025-101; Quality Biological) supplemented with 0.05 mM 2-mercaptoethanol (1610710; Bio-Rad), 10% FBS, and 1% penicillin/streptomycin. We have also checked the culture of THP-1 cells in complete DMEM, and we did not find any significant difference with respect to cell growth, cell morphology, or experimental outcomes. For the macrophage activation, RAW264.7 and THP-1 cells were treated with 0.3 μg/mL phorbol 12-myristate 13-acetate (PMA; P8139, Sigma-Aldrich) for 48 hr. Human monocytes were treated with 100 ng/mL M-CSF (216-MCC; R&D Systems) for 48 hr. To generate M1 and M2 macrophages, THP-1 cells activated by the PMA treatment were further treated with 100 ng/mL LPS (L2630, Sigma-Aldrich) or 20 ng/mL recombinant human interleukin-4 (200-04-20UG, PeproTech) for 48 hr, respectively. For time-lapse imaging of F-actin dynamics, RAW264.7 cells were transfected with LifeAct-GFP 1–2 days prior to the experiment.

### Cell plating

For experiments, cells were harvested at 80–90% confluency and plated on the microbead/SNS-immobilized Petri dish surface. According to the needs indicated in the manuscript, cells were detached either by trypsin or by a mild EDTA-based detaching solution (100 mL 10×HBSS + 10 mL 1 M HEPES + 10 mL 7.5% sodium bicarbonate + 2.4 mL 500 mM EDTA + 877.6 mL water; pH adjusted to 7.4). Cells were incubated in the detaching solution inside a CO_2_ incubator for 5–7 min. Next, cells were gently pipetted off from the culture flask, transferred into a 15 mL centrifuge tube, and centrifuged for 3 min at 300×*g*. The resulting cell pellet was resuspended in complete cell medium and transferred on microbead/SNS-immobilized glass surfaces. After incubation in a CO_2_ incubator for a certain time, the cell samples were ready for follow-up tests. All the experiments were performed using cells dispersed in complete growth medium, except experiments presented in Figure 4, in which serum-free medium was used instead.

### F-actin staining and immunostaining

For F-actin staining and immunostaining, cells were fixed with 4% formaldehyde (28908, Thermo Scientific) solution in PBS for 15 min at room temperature. Next, the formaldehyde solution was removed, and cells were permeabilized using 0.1% Triton X-100 (BP151-500, Fisher Scientific) solution in PBS for 20 min at room temperature. The cell samples were further blocked with 5 mg/mL BSA (9048-46-8, Fisher Scientific) solution in PBS for 45 min at room temperature. The samples were ready for the follow-up staining.

For F-actin staining, cell samples were treated with a 1:200 dilution of Alexa Fluor 488-labeled phalloidin (A12379, Invitrogen) and incubated for 30 min before imaging. For DNaseX immunostaining, the samples were first incubated with the primary antibody, anti-DNase1L1 (H00001774-MO2; Abnova), diluted 1:200 in PBS, for 1 hr at room temperature. Following this, the samples were incubated with a 1:200 dilution of Alexa Fluor 405-labeled secondary antibody (A31553, Invitrogen) in PBS for 1 hr at room temperature. After each incubation step, the samples were washed three times with PBS, with each wash involving a 5 min incubation.

### Pharmaceutical treatments

To induce GPI-linker cleavage, THP-1 macrophages were incubated with 2 U/mL and 5 U/mL of phospholipase C, phosphatidylinositol-specific from *Bacillus cereus* (P5542, Sigma-Aldrich), respectively, for 30 min. The cells were then plated and incubated on bead/SNS-coated surfaces for 1 hr prior to fixation and staining. For the inhibition of actin polymerization, Cytochalasin D (C8273, Sigma-Aldrich) and CK666 (182515, EMD Millipore) were added to the cell samples at concentrations of 1 μM and 100 μM, respectively. The cells were incubated with these inhibitors for 1 hr on the microbead/SNS-coated surfaces prior to fixation and staining for imaging.

### siRNA-mediated DNaseX knockdown

To perform the siRNA-mediated DNaseX knockdown, 4 µL of 10 µM siRNA (sc-77165, Santa Cruz) or scrambled siRNA (sc-37007, Santa Cruz) as negative control and 4 µL Lipofectamine RNAiMAX (100014472, Invitrogen) were added to 400 µL OptiMEM (11058-021, Gibco) and incubated for 15 min. In parallel, THP-1 cells at 70–80% confluency were detached, centrifuged, and resuspended in serum-free RPMI medium. The transfection cocktail solution was added to the cell suspension and incubated in a 35 mm Petri dish for 24 hr in a CO_2_ incubator. Afterward, the media of the transfected cells were replaced with complete RPMI medium supplemented with 0.3 μg/mL PMA. After 48 hr, the cells were ready for further tests.

### Preparation of bacterial biofilms

*S. aureus* bacterial strain was cultured to prepare bacterial biofilms. 5 mL LB broth was used to resuspend the freeze-dried bacterial powder in the vial (12600, ATCC). 50 µL of resuspended bacteria solution was added to 5 mL LB broth in a tube, which was incubated in a shaker-incubator overnight (~12 hr) at 37°C and 200 rpm shaking speed. Afterward, the cultured bacterial solution was amplified by diluting (1:10 vol/vol ratio) in the LB broth medium and further incubating inside the shaker-incubator (37°C and 200 rpm speed) for 4 hr.

For biofilm formation, the amplified *S. aureus* solution was added to TSB (Tryptic Soy Broth, 41298, Millipore) supplemented with 1% glucose at a 1:6 (vol/vol) ratio. 400 µL bacterial solution was incubated on a glass-bottom Petri dish (D35-14-1.5-N, Cellvis) at room temperature for 1–3 days. The biofilm thickness depends on the incubation times, with sporadic thin films formed in 1 day and large and thick films formed in 3 days. The Petri dish was kept in a pipette box with the bottom chamber filled with pure water to keep the bacterial solution from drying.

After biofilm formation, the surface was thoroughly washed with PBS three times to get rid of any unbound bacteria. The eDNA in the biofilm was stained with DITO-1 (502249754, Aat Bioquest) at 10 µM final concentration and 20 min incubation time at room temperature. The sample was subsequently washed with PBS three times. The biofilm was ready for further tests with macrophages.

### Macrophage incubation on bacterial biofilm

To study the interaction between *S. aureus* biofilm and macrophages, DITO-1-stained biofilm was treated with 0.25% Trypsin-EDTA (Corning; 25-053 CI) solution and incubated inside the 37°C incubator for 30 min. After incubation, the surface was washed with PBS three times. 200 µL Raw264.7 cell solution was added on the biofilm surface and incubated in a CO_2_ incubator at 37°C for 1 hr for static imaging. To perform live-cell imaging, RAW macrophage solution was plated directly onto the biofilm Petri dish mounted on the microscope. Raw macrophages were detached with mild detaching EDTA solution, centrifuged at 300 rcf for 3 min, and dispersed in culture medium for live-cell imaging.

### Microscopy and image processing

We performed imaging using a confocal microscope (Nikon Eclipse Ti2-E) with a 60× oil immersion objective. For real-time imaging, a lens heater was used to support cell viability. Laser beams at 405, 488, and 640 nm wavelengths were used as the excitation light sources. Image acquisition was conducted using the software (NIS-element) provided with the microscope.

### Quantification and statistical analysis

Data were analyzed using MATLAB codes developed in our lab. Results were reported as mean ± standard deviation (SD). The results of key experiments were repeated three times, and data were pooled together for statistical analysis. The volume of sample analyzed is specified in the figure legends. All the data were analyzed using ImageJ or MATLAB codes, and plotted using GraphPad Prism. t-Tests were conducted using GraphPad Prism. For all statistical tests shown: ns: non-significant, *p<0.05, **p<0.01, ***p<0.001, ****p<0.0001.

## Data Availability

All data supporting the findings of this study are included within the article and its supplementary files. Figures 1 to 7–source data contain the numerical data and statistical analysis used to generate the plots in Figures 1—7 and associate supplement figures. Original microscopy images and videos are available online https://doi.org/10.6084/m9.figshare.34176180. The following dataset was generated: WangX 2026Original microscopy images and videosfigshare10.6084/m9.figshare.34176180

## Additional files

MDAR checklist
