# Supplementary material for: Early recruitment of membrane-bound DNaseX to phagocytic cups in macrophages
Source: eLife. 2026 Oct 8;15:RP110907. doi: 10.7554/eLife.110907 (PMC13649258; doi:10.7554/eLife.110907)
Supplement: Peer review history

# Editor report: eLife Assessment

**DOI:** 10.7554/eLife.110907.3.sa0

**Authors:** Larissa D Cunha

**Affiliations:** University of Sao Paulo Brazil

**Keywords:** Solid, Valuable

This work by Pyne and Pandey et al. addresses DNase X (DNase1L1) activity at the macrophage phagocytic cup, using an innovative imaging approach that couples visualization of cup formation to spatially resolve DNA degradation. The methodology is technically sound, and the central finding that DNA digestion begins prior to phagolysosomal maturation is well supported. Overall, the study is **solid** and provides a **valuable** framework for investigating early events at the phagocytic cup that may shape responses to pathogens and inflammatory disease.

# Referee report: Reviewer #1 (Public review)

**DOI:** 10.7554/eLife.110907.3.sa1

Pyne and Pandey et al. report the observation of early DNA degradation at the phagocytic cup during macrophage engulfment. Using an elegant experimental system that combines actin staining to visualise cup formation with direct monitoring of DNA degradation, the authors identify rapid recruitment of the membrane-bound nuclease DNase X (DNase1L1) to nascent phagocytic cups. This recruitment occurs within minutes of cup formation, is independent of DNA presence at the substrate, and appears to originate from intracellular membrane structures rather than from the extracellular environment. The results support the conclusion that DNase X activity is present at the phagocytic cup and that DNA digestion can begin prior to phagolysosomal maturation.

The study is technically strong. The experimental system is clean, specific, and allows precise spatial and temporal detection of DNA degradation. The imaging-based approaches are carefully executed and enable convincing visualisation of DNase X recruitment and activity. The use of an alternative substrate beyond the primary SNS system strengthens the core observation, and the data broadly support the authors' central claim.

However, several limitations temper the physiological interpretation. The system relies largely on short, free DNA substrates, leaving open how efficiently DNase X processes more complex or physiologically relevant DNA structures, such as nucleosome-bound DNA or neutrophil extracellular traps (NETs). It remains unclear whether DNase X deficiency would alter macrophage responses to larger nucleic acid structures, influence engulfment efficiency, or modify downstream inflammatory signalling pathways such as TLR9 or STING activation. Moreover, the experimental setup prevents full phagocytic cup closure, potentially prolonging DNase activity compared with physiological phagocytosis, which typically proceeds rapidly to cargo internalisation. For example, the peak signal observed in Figure 5 occurs approximately 90 minutes after phagocytic cup formation, a time point at which many phagocytic cups would be expected to have already closed under physiological conditions. Additional work using fully engulfed cargo in more physiological contexts would clarify whether early DNase X activity meaningfully contributes to overall DNA clearance kinetics.

Mechanistically, the signal that triggers DNase X recruitment remains unresolved. Although actin rearrangement was excluded as the primary driver, the upstream cues that direct DNase X-containing membrane structures to the forming cup are not yet defined.

In the broader context, early DNase X activity at the phagocytic cup could represent an additional safeguard against inflammatory signalling by limiting extracellular or surface-associated DNA before phagolysosomal degradation by DNase II. This mechanism may be particularly relevant in settings where DNA fragmentation before engulfment is incomplete, such as necroptosis or NET formation. Determining whether DNase X deficiency exacerbates inflammatory responses, alters DNA clearance efficiency in vivo, or contributes to immune pathology will be critical for establishing its physiological and disease relevance.

Overall, this is a compelling study that introduces a novel concept of pre-phagolysosomal DNA digestion. The conclusions are well supported within the in vitro system used, but further investigation using diverse DNA substrates and physiologically relevant models will be required to fully define the impact of this mechanism on immune regulation and disease.

Comments on revised version.

The authors have responded constructively to the points raised in my original review. Fig. S5 extends the substrate range beyond the 18-bp SNS construct by showing degradation of plasmid DNA immobilised on microbeads, and fig. S8 addresses my concern that the surface-immobilised platform prevents phagocytic cup closure, by demonstrating DNase activity at cups forming over free, internalisable beads. Together with the live-imaging data in Fig. 1I, these additions support the central claim that DNase activity begins at the nascent phagocytic cup, before closure and well before phagolysosomal maturation. I consider my principal concerns addressed and see no reason to alter the eLife Assessment.

One point remains for readers rather than as a request. The new data establish that DNA at the cup is degraded, but do not themselves demonstrate that DNaseX is responsible, since neither fig. S5 nor the biofilm experiments in Fig. 7 include the PI-PLC or siRNA controls used in Fig. 3; the authors appropriately acknowledge this limitation for the biofilm data. The physiological questions raised in my original review - the handling of chromatin-associated DNA and NETs, and whether DNaseX activity at the cup limits downstream sensing of extracellular DNA - remain open, and the manuscript is appropriately framed as establishing the phenomenon rather than resolving its physiological role.

# Referee report: Reviewer #2 (Public review)

**DOI:** 10.7554/eLife.110907.3.sa2

Summary:

This manuscript presents an elegant and innovative imaging approach to visualize DNase activity at the interface between macrophages and extracellular substrates. The platform is technically strong and enables the study of localized DNA degradation with high spatial resolution. The work is of clear interest and provides a useful framework to investigate how immune cells process extracellular DNA. However, several aspects of the mechanistic interpretation and conceptual framing would benefit from clarification.

Strengths:

(1) The study introduces a creative and well-designed imaging platform that allows visualization of localized DNase activity at cell-substrate interfaces.

(2) The approach is technically robust and represents a valuable tool that could be broadly useful to the field.

(3) The experiments are thoughtfully designed and address an important question regarding how immune cells interact with extracellular DNA.

(4) The work opens interesting avenues for studying DNA processing in contexts such as infection and inflammation.

Comments on revised version.

The authors have carefully addressed the points raised in my previous review, and the manuscript has been revised accordingly. I am satisfied with the revisions and have no further comments at this stage.

# Author comment: Author response

**DOI:** 10.7554/eLife.110907.3.sa3

**Authors:** Arghajit Pyne, Vivek Pandey, Subhankar Kundu, Sachie Ikegami, Xuefeng Wang

**Affiliations:** University of Cincinnati Cincinnati United States; University of Cincinnati Cincinnati United States; University of Cincinnati Cincinnati United States; University of Cincinnati Cincinnati United States; University of Cincinnati Cincinnati United States

The following is the authors’ response to the original reviews.

**eLife Assessment**

This work by Pyne and Pandey et al. addresses DNase X (DNase1L1) activity at the macrophage phagocytic cup, using an innovative imaging approach that couples visualization of cup formation to spatially resolve DNA degradation. The methodology is technically sound, and the central finding that DNA digestion begins prior to phagolysosomal maturation is considered well supported, though some mechanistic claims may benefit from further evidence and more cautious framing. Overall, the study is solid and provides a valuable framework for investigating early events at the phagocytic cup that may shape responses to pathogens and inflammatory disease.

Thanks for the overall review.

**Public Reviews:**

**Reviewer #1 (Public review):**

Pyne and Pandey et al. report the observation of early DNA degradation at the phagocytic cup during macrophage engulfment. Using an elegant experimental system that combines actin staining to visualise cup formation with direct monitoring of DNA degradation, the authors identify rapid recruitment of the membrane-bound nuclease DNase X (DNase1L1) to nascent phagocytic cups. This recruitment occurs within minutes of cup formation, is independent of DNA presence at the substrate, and appears to originate from intracellular membrane structures rather than from the extracellular environment. The results support the conclusion that DNase X activity is present at the phagocytic cup and that DNA digestion can begin prior to phagolysosomal maturation.

The study is technically strong. The experimental system is clean, specific, and allows precise spatial and temporal detection of DNA degradation. The imaging-based approaches are carefully executed and enable convincing visualisation of DNase X recruitment and activity. The use of an alternative substrate beyond the primary SNS system strengthens the core observation, and the data broadly support the authors' central claim.

Thanks for the positive comments.

However, several limitations temper the physiological interpretation. The system relies largely on short, free DNA substrates, leaving open how efficiently DNase X processes more complex or physiologically relevant DNA structures, such as nucleosome-bound DNA or neutrophil extracellular traps (NETs). It remains unclear whether DNase X deficiency would alter macrophage responses to larger nucleic acid structures, influence engulfment efficiency, or modify downstream inflammatory signalling pathways such as TLR9 or STING activation.

Thanks for the comments. It is a constructive suggestion to test how DNase X in phagocytic cups (PCs) may degrade long DNA or chromosomal DNA, as the current experiment setup mainly used 18bp DNA as the DNase sensor construct. Now we included new data of DNase response of macrophage to plasmid DNA (extracted from *E. coli*) immobilized on microbead surface. The result show that macrophage also degrade the plasmid DNA, suggesting that the DNase in PC is rather versatile and can degrade long DNA. This result is included as fig. S5.

It would be certainly interesting to explore the role of DNase X in phagocytic efficiency and downstream inflammatory pathways. While these could be the future research directions, the goal of current manuscript is to demonstrate a new DNase activity in PCs and calibrate its several core features (temporal and spatial dynamics and response to biofilm, etc.). Expanding the topic further will require substantial resource and effort, and may make the current manuscript bloated with data. Therefore, we didn’t pursue the study of DNase of PCs in the context of phagocytosis efficiency and downstream pathways.

Moreover, the experimental setup prevents full phagocytic cup closure, potentially prolonging DNase activity compared with physiological phagocytosis, which typically proceeds rapidly to cargo internalisation. For example, the peak signal observed in Figure 5 occurs approximately 90 minutes after phagocytic cup formation, a time point at which many phagocytic cups would be expected to have already closed under physiological conditions.

Additional work using fully engulfed cargo in more physiological contexts would clarify whether early DNase X activity meaningfully contributes to overall DNA clearance kinetics.

Real-time imaging of SNS signal and F-actin in macrophages, shown in Fig. 1I in the manuscript, suggests that DNase activity in PCs appear within one minute after PC formation. We do resonate with the reviewer’s concern about the experiment platform used in this work, namely, the microbeads are immobilized on the glass surface, preventing the natural closure of PCs. This artificial design may create biased observation of DNase activity during phagocytosis.

In light of this concern, we developed a new assay by using free SNS-coated microbeads (non-immobilized). These beads can be completely internalized by macrophages through phagocytosis. During experiments, we searched for the PCs just starting to form over free SNS-coated beads. Despite the rare occurrence of these events (phagocytosis process has a rather short time windows), we did find the evidence that PCs already exhibit DNase activity before their closures, as shown in fig. S8.

Accordingly, we added one paragraph to the main text, “Because surface-immobilized microbeads prevent PC closure, they likely prolong PC formation and may alter the observed temporal dynamics of DNase activity within the PC. To determine whether the PC exhibits DNase activity before closure, we prepared SNS-coated free microbeads and fed them to adherent macrophages. The results showed that PCs indeed exhibited DNase activity before cup closure in response to free microbeads (fig. S8), confirming that DNase activity is initiated rapidly during PC formation.”

Mechanistically, the signal that triggers DNase X recruitment remains unresolved. Although actin rearrangement was excluded as the primary driver, the upstream cues that direct DNase X-containing membrane structures to the forming cup are not yet defined.

We agree that the biochemical signals responsible for initiating DNase X recruitment remain unresolved. Despite our efforts to identify such signals by exposing macrophages to a variety of immunogenic stimuli, we consistently observed DNase X recruitment to phagocytic cups under all conditions tested. Based on these findings, we conclude that DNase X recruitment to phagocytic cups is constitutive rather than stimulus-dependent.

Nevertheless, even if this recruitment is constitutive, it is likely that an intracellular biochemical cue initiates or regulates the recruitment process. Unfortunately, we have not yet been able to identify this upstream signal. We have acknowledged this limitation and included this consideration in the Discussion as an important direction for future investigation.

In previous studies, we also examined DNase activity at phagocytic cups following inhibition of the cGAS-STING and TLR9 pathways, two major cellular DNA-sensing mechanisms. However, inhibition of either pathway did not alter DNase activity at phagocytic cups. As these experiments yielded negative results and did not provide mechanistic insight into DNase X recruitment, we did not include them in the current manuscript.

In the broader context, early DNase X activity at the phagocytic cup could represent an additional safeguard against inflammatory signalling by limiting extracellular or surface-associated DNA before phagolysosomal degradation by DNase II. This mechanism may be particularly relevant in settings where DNA fragmentation before engulfment is incomplete, such as necroptosis or NET formation. Determining whether DNase X deficiency exacerbates inflammatory responses, alters DNA clearance efficiency in vivo, or contributes to immune pathology will be critical for establishing its physiological and disease relevance.

Thanks for the constructive comments which provide valuable suggestion for the future work.

Overall, this is a compelling study that introduces a novel concept of pre-phagolysosomal DNA digestion. The conclusions are well supported within the in vitro system used, but further investigation using diverse DNA substrates and physiologically relevant models will be required to fully define the impact of this mechanism on immune regulation and disease.

We appreciate the insightful comments and agree that *in vivo* experiments would provide valuable information for future studies. The current manuscript focuses on a cellular function identified using an *in vitro* experimental system. The results suggest that DNaseX may play important physiological roles in immune defense and extracellular DNA clearance, highlighting the need for further investigation using animal models or other *in vivo* systems. These are exciting directions that we are actively planning to pursue in our future research.

We are not able to incorporate *in vivo* studies into the current revision, as such experiments would require the establishment of fundamentally different experimental models, the acquisition of new technical expertise, and substantial additional financial support. These efforts are beyond the scope and timeline of the present manuscript revision. We believe that the current *in vitro* findings provide a foundation for these future *in vivo* investigations.

**Reviewer #2 (Public review):**

Summary:

This manuscript presents an elegant and innovative imaging approach to visualize DNase activity at the interface between macrophages and extracellular substrates. The platform is technically strong and enables the study of localized DNA degradation with high spatial resolution. The work is of clear interest and provides a useful framework to investigate how immune cells process extracellular DNA. However, several aspects of the mechanistic interpretation and conceptual framing would benefit from clarification.

Strengths:

(1) The study introduces a creative and well-designed imaging platform that allows visualization of localized DNase activity at cell-substrate interfaces.

(2) The approach is technically robust and represents a valuable tool that could be broadly useful to the field.

(3) The experiments are thoughtfully designed and address an important question regarding how immune cells interact with extracellular DNA.

(4) The work opens interesting avenues for studying DNA processing in contexts such as infection and inflammation.

Thanks for the positive comments.

Weaknesses:

While the experimental approach is strong, several key conclusions rely on interpretations that would benefit from further clarification:

(1) First, the conclusion that DNaseX is recruited to phagocytic cups from the "cytoplasm" appears conceptually imprecise. Given that DNaseX is a membrane-anchored protein, it is unlikely to exist as a freely soluble cytoplasmic pool. A more plausible interpretation is that DNaseX is supplied from intracellular membrane compartments. This interpretation would also be more consistent with the data showing dependence on a membrane anchor.

We thank the reviewer for this insightful comment. We agree that, as a GPI-anchored protein, DNaseX is likely transported from the endoplasmic reticulum and Golgi apparatus to the plasma membrane via secretory vesicles carrying the protein. The original subtitle “DNaseX in PCs is recruited from the cytoplasm, not from the plasma membrane” in the manuscript, is misleading, as it may imply that DNaseX is a cytoplasmic soluble protein. Our original point is to state that DNaseX is recruited intracellularly, not from the adjacent cell membrane domain. To avoid the ambiguity, we have revised that subtitle to “DNaseX in PCs is recruited intracellularly, not from the plasma membrane”. In the revision, we also emphasized the that DNaseX is likely delivered by the vesicles, which were observed in Fig. S13.

(2) Second, the interpretation that actin polymerization is not required for DNaseX recruitment raises concerns. Phagocytic cup formation is known to depend strongly on actin dynamics, and it is therefore unclear whether the structures observed under actin inhibition represent fully formed functional cups or partial cell-substrate contacts. This distinction is important for interpreting recruitment versus activity, particularly since enzymatic activity is reduced under these conditions.

We appreciate the reviewer’s comment and agree with this point. The data presented in the manuscript demonstrate the relationships, but not causalities, among F-actin signal, DNaseX signal (detected by immunostaining), and SNS signal (reflecting DNase activity) in PCs. Specifically, our results show a strong correlation between the F-actin and SNS signals, whereas the correlation between the F-actin and DNaseX signals is weak. These correlations do not warrant the causality relations.

Experimentally establishing causality is challenging because F-actin is an essential structural component of the phagocytic cup and cannot be completely eliminated from PCs. To ensure rigorous interpretation of the data, we have revised the manuscript to present the findings more cautiously and avoid implying a causal relationship where it has not been experimentally demonstrated.

We revised the section title: “Actin polymerization is dispensable for DNaseX recruitment but essential for DNase activity in the PCs” to “Actin polymerization is correlated with DNase activity in PCs, but not with DNaseX recruitment”. We also revise the abstract and the main text correspondingly to reflect this point.

(3) Third, the identification of DNaseX as the main nuclease responsible for the observed activity is not fully resolved. The conclusions rely primarily on gene silencing and staining approaches, but the specificity of these strategies relative to other nucleases is not addressed. It therefore remains possible that additional enzymes contribute to the observed activity.

The reviewer raises an important point. We acknowledge that gene silencing and immunostaining may not be entirely specific, as they could potentially affect or detect other members of the DNase family that share structural similarities with DNaseX. Indeed, the official name of DNaseX, Deoxyribonuclease-1-like 1, suggests a close structural relationship with DNase I.

Our conclusion regarding the specificity of DNaseX is based on the combined evidence from multiple independent approaches rather than any single experiment. In addition to gene silencing and immunostaining, we demonstrated that cleavage of the GPI anchor with PI-PLC markedly reduced or completely abolished DNase activity in PCs. This experiment provides particularly strong evidence because DNaseX is the only known membrane-bound DNase that is anchored to the plasma membrane via a GPI linker.

Taken together, the complementary results from GPI-anchor cleavage, gene silencing, and immunostaining provide convergent evidence supporting the conclusion that DNaseX is the primary enzyme responsible for the DNase activity observed in PCs. We therefore believe that the combined data provide sufficient specificity to support our conclusion.

(4) Finally, the interpretation of the biofilm experiments may be overstated. While the data clearly show localized DNA degradation in contact with macrophages, it is not fully established that this process depends specifically on phagocytic cup structures. An alternative explanation is that membrane-associated DNase activity more generally mediates this effect. In addition, the physiological relevance of this mechanism would benefit from further discussion.

We agree with the reviewer on this point. Although our data demonstrate that macrophages degrade eDNA in biofilms through direct physical contact, they do not establish that phagocytic cups are the primary structures responsible for mediating this process.

We attempted to obtain direct evidence by simultaneously imaging F-actin structures in macrophages and eDNA degradation within biofilms, with the goal of visualizing phagocytic cup formation around eDNA filaments. However, this proved to be technically challenging. Unlike a flat glass surface, biofilms possess substantial thickness and an uneven three-dimensional topology, making it difficult to achieve high-resolution imaging of F-actin structures with sufficient clarity. In addition, eDNA filaments, unlike microparticles, may not induce the formation of canonical F-actin-rich phagocytic cups. Consequently, we were unable to obtain convincing imaging data demonstrating that phagocytic cups are the structures responsible for eDNA degradation within biofilms.

To ensure a rigorous interpretation of our findings, we have revised the manuscript to avoid overstating this conclusion and to more accurately reflect the limitations of the current data. We added this paragraph in the revision: “While the results showed that macrophages degrade eDNA in biofilms through physical contact, it has not been confirmed that this degradation is mediated by DNaseX within the PC, as eDNA filaments are not expected to induce the formation of a typical PC structure. An alternative possibility is that DNaseX localized on the cell membrane, including within the plaque-like clusters (fig. S13), comes into direct contact with eDNA and mediates its degradation through physical contact.”

Overall, the study is technically strong and introduces a valuable methodology, but several central conclusions are only partially supported by the current data and would benefit from more cautious interpretation and clearer conceptual framing.

**Recommendations for the authors:**

**Reviewer #1 (Recommendations for the authors):**

The data are clearly presented, and the manuscript is well written, making it a pleasure to read.

**Reviewer #2 (Recommendations for the authors):**

(1) Clarify the description of DNaseX origin by replacing "cytoplasmic" with more precise terminology (e.g., intracellular membrane compartments), and revise the interpretation accordingly.

Revision has been made according to the reviewer’s suggestion. Please see the point-to-point response.

(2) Provide a clearer definition of what constitutes a phagocytic cup under actin inhibition conditions, and discuss whether the observed structures represent fully functional compartments.

Revision has been made according to the reviewer’s suggestion. Please see the point-to-point response.

(3) Consider including additional controls or discussion addressing the specificity of the gene silencing approach, particularly in relation to other nucleases. If feasible, assess the potential contribution or localization of other nucleases (immunofluoresce for DNase I, for example), or explicitly acknowledge this as a limitation.

Revision has been made according to the reviewer’s suggestion. Please see the point-to-point response.

(4) Refine the interpretation of the biofilm experiments to distinguish between phagocytic cup-specific mechanisms and more general membrane-associated activity. Expand the discussion on the physiological relevance of the findings, particularly in the context of complex environments such as biofilms or extracellular DNA structures in vivo.

Revision has been made according to the reviewer’s suggestion. Please see the point-to-point response.

(5) Minor: Improve consistency in terminology throughout the manuscript (e.g., membrane-associated vs cytoplasmic localization) to avoid conceptual confusion.

We revised all “membrane-associated” to “membrane-bound” for the consistency of terminology. The confusion due to the use of “cytoplasmic” has been rectified. Please see the point-to-point response.
